# Influenza NS1 directly modulates Hedgehog signaling during infection

**DOI:** 10.1371/journal.ppat.1006588

**Published:** 2017-08-24

**Authors:** Margery G. Smelkinson, Annabel Guichard, John R. Teijaro, Meghana Malur, Maria Eugenia Loureiro, Prashant Jain, Sundar Ganesan, Elina I. Zúñiga, Robert M. Krug, Michael B. Oldstone, Ethan Bier

**Affiliations:** 1 Section of Cell and Developmental Biology, University of California, San Diego, La Jolla, California, United States of America; 2 Immunology and Microbial Science, Scripps Research Institute, La Jolla, California, United States of America; 3 Department of Molecular Biosciences, University of Texas at Austin, Austin, Texas, United States of America; 4 Division of Biological Sciences, University of California San Diego, La Jolla, California, United States of America; 5 Instituto de Ciencia y Tecnología Dr. César Milstein - CONICET, Saladillo, Argentina; 6 Biological Imaging Section, Research Technologies Branch, National Institute of Allergy and Infectious Diseases, National Institutes of Health, Bethesda, Maryland, United States of America; University of Chicago, UNITED STATES

## Abstract

The multifunctional NS1 protein of influenza A viruses suppresses host cellular defense mechanisms and subverts other cellular functions. We report here on a new role for NS1 in modifying cell-cell signaling via the Hedgehog (Hh) pathway. Genetic epistasis experiments and FRET-FLIM assays in *Drosophila* suggest that NS1 interacts directly with the transcriptional mediator, Ci/Gli1. We further confirmed that Hh target genes are activated cell-autonomously in transfected human lung epithelial cells expressing NS1, and in infected mouse lungs. We identified a point mutation in NS1, A122V, that modulates this activity in a context-dependent fashion. When the A122V mutation was incorporated into a mouse-adapted influenza A virus, it cell-autonomously enhanced expression of some Hh targets in the mouse lung, including IL6, and hastened lethality. These results indicate that, in addition to its multiple intracellular functions, NS1 also modifies a highly conserved signaling pathway, at least in part via cell autonomous activities. We discuss how this new Hh modulating function of NS1 may influence host lethality, possibly through controlling cytokine production, and how these new insights provide potential strategies for combating infection.

## Author summary

The NS1 protein produced by influenza A viruses alters host cellular defense mechanisms. We report here on a new role for NS1 in modifying cell-cell communication via the Hedgehog (Hh) signaling pathway. Genetic and microscopy studies in flies indicate that NS1 alters the transcriptional read-out of Hh targets by interacting directly with the transcriptional effector, Ci/Gli. Infected mouse lungs and human lung cells transfected with NS1 also revealed an upregulation of Hh target genes, including the non-canonical target IL6. We identified a point mutation in NS1 (A122V) that impairs this target activation in *Drosophila*. The A122V mutation was incorporated into a mouse-adapted influenza A virus and was found to cell-autonomously increase expression of some Hh targets and hastened lethality in mice. NS1 also exhibited A122V-dependent effects on BMP target gene expression in a human lung cell line. These results indicate that, in addition to its multiple intracellular functions, NS1 also modifies communication between host cells. This new activity of NS1 appears to influence lethality caused by the virus and may facilitate viral dissemination by extending host survival.

## Introduction

The multifunctional Non-Structural-1 protein (NS1) is one of 14 proteins encoded by influenza A virus [1–3]. NS1 interacts with several viral and host components to subvert cellular defense mechanisms and promote viral replication via two domains: an N-terminal RNA-binding domain (RBD) and an effector domain (ED), both of which can multimerize. The RBD binds dsRNA, a common viral replication intermediate, thereby preventing dsRNA-mediated activation of a key interferon-induced antiviral activity [4]. The ED sequesters multiple host proteins required for cellular mRNA maturation as well as export factors controlling apoptosis [2]. The functions of the NS1 protein benefit the virus by inhibiting the production and activation of host antiviral factors, establishing preferential viral mRNA translation, and extending host cell viability to allow sufficient viral maturation.

Most interactions between the influenza NS1 protein and its host targets have been identified in cells grown in culture for which the assays are typically cell autonomous (e.g., viral replication, altered host gene expression). We sought to identify novel targets of NS1 that might be involved in cell-cell communication using *Drosophila melanogaster*, a highly developed genetic system for analyzing well-conserved signaling pathways [5,6] and for elucidating interactions between host and pathogen encoded proteins [7,8], including viral proteins from influenza [9]. Using this system, we have identified a novel activity of the NS1 protein involved in regulating the Hedgehog (Hh) signaling pathway.

The Hh signaling pathway plays a central role in growth and development [10,11], tissue repair [12], and adult immune responses [13–15] of vertebrates and invertebrates. In the *Drosophila* wing primordium (or wing imaginal disc), Hh is secreted from cells in the posterior compartment and binds to the Patched (Ptc) receptor, resulting in phosphorylation and surface accumulation of the seven pass transmembrane domain protein Smoothened (Smo) in a stripe of cells in the anterior compartment (referred to as the central organizer) [16]. Activated Smo, in turn, recruits Costal-2 (Cos-2) to the plasma membrane, disrupting an inhibitory complex with the transcription factor Cubitus interruptus (Ci; Gli in mammals), thereby stabilizing and activating the full-length Ci-155 protein. In the absence of Hh signaling, microtubule associated Cos-2 promotes Ci-155 phosphorylation via cAMP-dependent Protein Kinase A (PKA) and other kinases, resulting in partial proteolysis of Ci-155 to a N-terminal repressor (Ci-75) that silences a subset of Hh target genes [16].

In the current study, we report that NS1 alters expression of Hh target genes by directly modulating the specific activity of the transcriptional effector, Ci/Gli. This novel signaling activity remains unaltered by previously defined mutations in NS1 that block its interactions with known host effectors. We identified a novel point mutation in a surface residue of NS1 (A122V), however, that does abrogate this signaling function. Incorporation of the A122V mutation into a mouse-adapted influenza virus increased expression of some Hh targets and cytokines, accelerated lethality, and increased host morbidity relative to the parental virus. These effects of NS1 are at least in part due to direct cell autonomous effects of NS1 since transfection of NS1 alone into human lung cell lines altered expression of BMP2, the mammalian homologue of *Drosophila dpp*, in an A122V-dependent fashion. These findings reveal a new signaling function and binding domain of NS1 that modulates influenza virulence and potentially provides new therapeutic avenues for treating infection.

## Results

### NS1 alters a branch of Hh signaling in *Drosophila*

**Fig 1:**
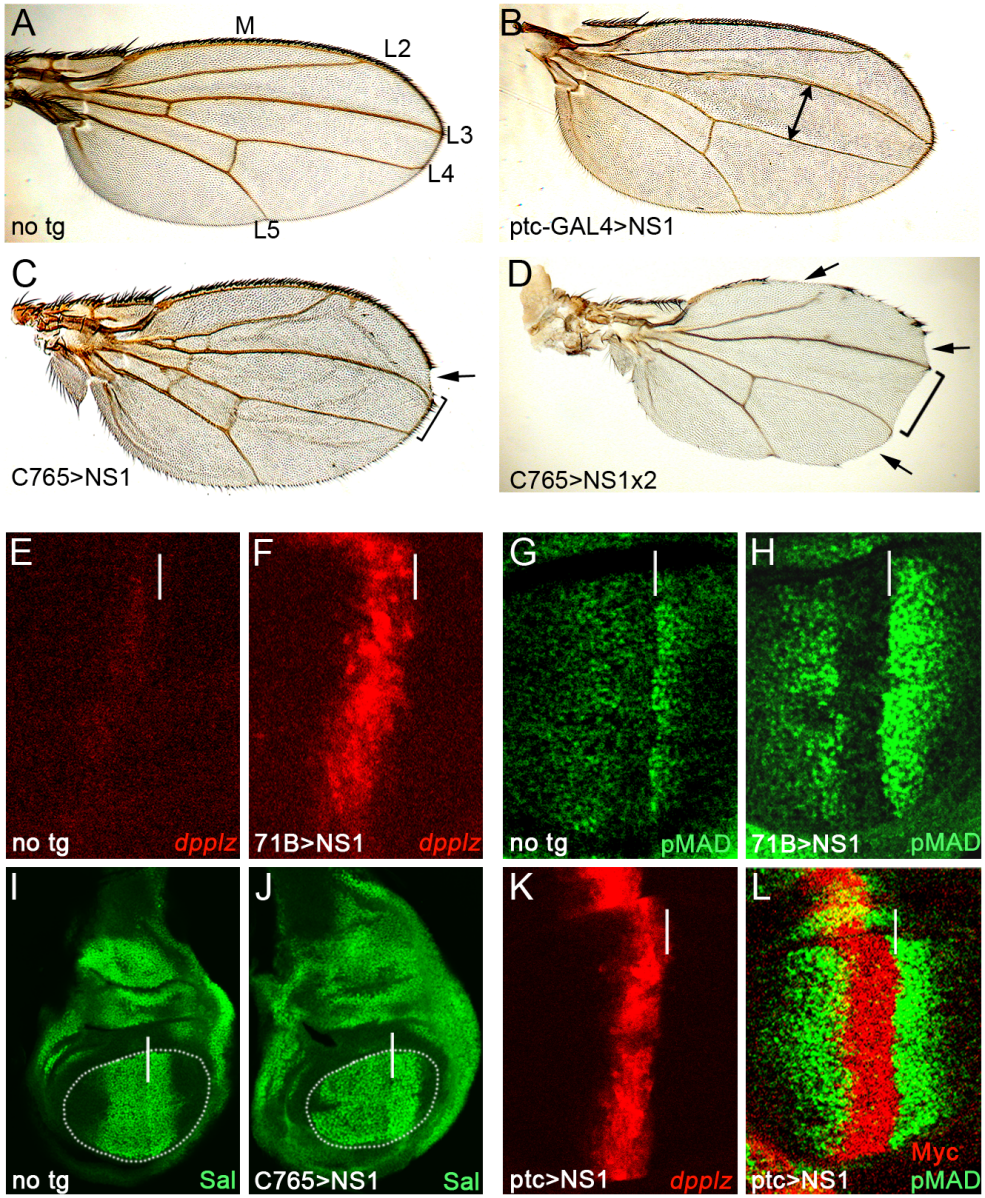
NS1 enhances *dpp* expression and Dpp signaling in the fly. (A) A wing with no transgene (no tg) with demarcated longitudinal veins L2-L5, M = margin. (B, C) The distance between the L3 and L4 veins increases in wings expressing NS1 along the A/P border with the ptc-GAL4 driver (B, double arrow) or the ubiquitous C765-GAL4 driver (C), a phenotype exacerbated by an additional copy of NS1 (D) (brackets in C and D). In C and D, notches are also present along the margin (arrows). Wing imaginal discs with no transgene (E,G,I) or discs expressing NS1 under the control of the ubiquitous 71B-GAL4 (F, H), C765-GAL4 (J), or ptc-GAL4 (K,L) drivers stained with antibodies to visualize *dpp-lacZ* expression (E,F,K), phospho-Smad1(G,H,L), Spalt (I, J), or the Myc epitope tag on NS1 (L). Ubiquitous expression of NS1 leads to elevated expression of *dpp-lacZ* (F vs. E), stronger and broadened pMAD expression (H vs. G), and expanded expression of the Dpp target gene Spalt (J vs. I, wing pouch outlined). Restricted expression of NS1 along the A/P border (detected by the C-terminal Myc tag) also results in enhanced *dpp-lacZ* expression (K, vs. E) and pMAD staining in cells neighboring the A/P border (L vs. G). In all figures of wing discs, dorsal is up, anterior is to the left. White lines indicate the A/P border.

We expressed an NS1 cDNA from the A/Vietnam/1203/04 H5N1 viral strain (referred to as NS1(Vn)), double tagged with HA (N-terminus) and Myc (C-terminus) epitopes in the *Drosophila* wing by placing it under the transcriptional control of the yeast upstream activating sequence (UAS) [17]. Flies carrying this construct were crossed to strains expressing the yeast GAL4 transactivator protein in wing-specific patterns to conditionally activate expression of the UAS-NS1(Vn) transgene in the wings of progeny (Fig 1B–1D). Localized expression of NS1(Vn) in the central organizer increased the distance between wing veins L3 and L4 1.34X compared to wings with no transgene (Fig 1A and 1B, n = 5–7, p<1.6x10^-5^). Similarly, ubiquitous expression throughout the wing increased the distance between the L3 and L4 veins 1.3X (Fig 1C, n = 3–7, p<7.4x10^-5^) in the presence of one copy of NS1 and 1.47X (Fig 1D, n = 6–7, p<2.35x10^-6^) in the presence of two copies, a phenotype indicative of spatially broadened Hh signaling [18,19]. Notches along the edge of the wing were also observed (arrows in Fig 1C and 1D) indicating that NS1 has additional non-Hh related effects, which may be mediated by the Wg or Notch signaling pathways (see S1 Text).

Consistent with its adult wing phenotype, expression of NS1(Vn) in ubiquitous (Fig 1F) or organizer-specific (Fig 1K) patterns increased expression of a minimal synthetic *dpp-lacZ* reporter [20], a known Hh target, 7.48X (n = 3, p<2.8x10^-3^) along the A/P border in late larval wing discs. In contrast, expression of other Hh target genes, such as Ptc, Collier (Col), and Engrailed (En), did not appear appreciably altered by NS1(Vn) (S1A, S1B, S1E, S1F, S1I and S1J Fig), suggesting that this effect was limited to a specific subset of Hh targets genes (see below, however, regarding the extended effects of the more active NS1 protein from the PR8 strain of influenza).

Dpp secreted from the central organizer diffuses to form a long range gradient extending both anteriorly and posteriorly [21], which can be detected *in situ* by antibody staining for the phosphorylated (and active) form of the cytoplasmic signal transducer Mothers against decapentaplegic (pMAD) [22] or Dpp target genes such as *spalt* [23,24]. Both ubiquitous (Fig 1G–1J) and central-organizer specific (Fig 1K and 1L) expression of NS1(Vn) in the wing disc intensified pMAD staining 2.36X (n = 4–6, p<0.025) and broadened the domain of Spalt staining (1.3x distance from A/P, n = 3–5, p<0.03) indicating an expanded range of Dpp signaling. Consistent with NS1(Vn) acting non-autonomously via Dpp on adjacent cells, preventing Dpp diffusion out of the central organizer abolished this induction of BMP signaling (S1 Text and S2 Fig).

### Amino acid residue A122 of NS1 is required for its Hh signaling modulating activity

**Fig 2:**
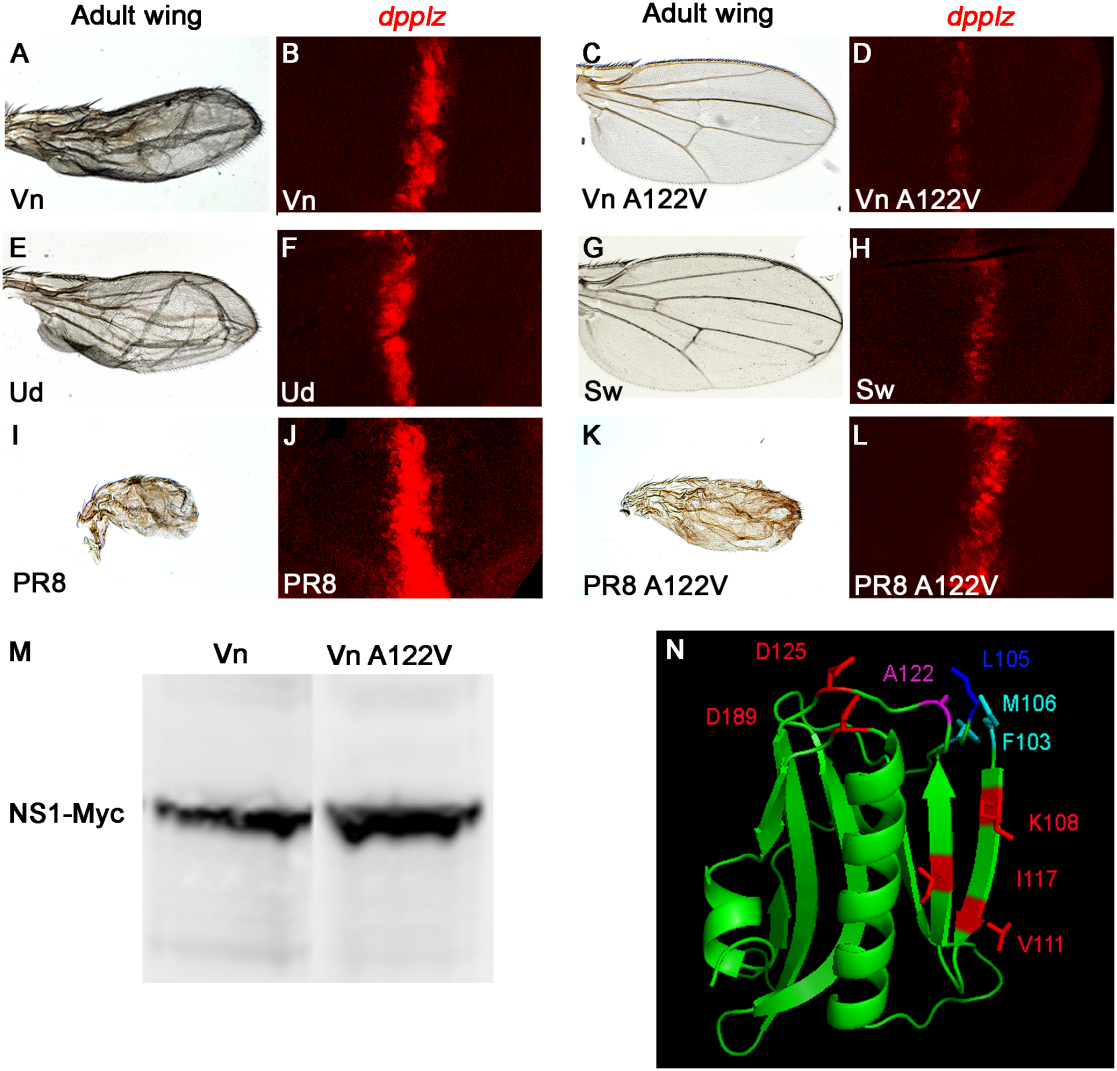
A point mutation in NS1 reduces its ability to activate *dpp-lacZ* expression. NS1 transgenes from different viral strains were expressed in adult wings with the strong MS1096-GAL4 driver (A, C, E, G, I, K) or in *dpp-lacZ* expressing wing discs with the moderate ubiquitious 71B-GAL4 driver (B, D, F, H, J, L). The NS1(Vn) phenotype (A,B) was greatly reduced by the A122V mutation (C,D). NS1(Ud) has a phenotype similar to NS1(VN) (E,F), whereas NS1(Sw) has weaker (G,H) and NS1(PR8) stronger (I,J) activity. The NS1(PR8) phenotype is significantly rescued by the A122V mutation (K,L). (M) Equivalent protein levels of the NS1(Vn) and NS1(Vn)-A122V were detected on Western blots of fly extracts using an antibody to C-terminal Myc tags. (N) Structure of the ED of NS1(Vn) displaying the position of A122 (magenta), L105 (blue), and surface residues altered in NS1(Sw) (red), or NS1(PR8) (cyan) (see Table A in S1 Text for results).

Since NS1 is a multifunctional effector protein that interacts with several well-characterized host factors, we next examined the role of known mutations that disrupt such interactions. Surprisingly, none of the mutations in NS1 residues that abrogate its established interactions with host effectors compromised its Hh-modulating activity in the *Drosophila* wing (see S1 Text, S3 Fig, and Table A in S1 Text for further analysis), nor did co-expression of NS1 with candidate effectors such as PI3K or polyA binding protein (see Table A in S1 Text). We, therefore, conducted an unbiased mutagenesis screen to isolate new mutant alleles of NS1 that could no longer perform this novel signaling function. One revertant allele we recovered greatly reduced the NS1 wing phenotype as well as reduced NS1-dependent expression of *dpp-lacZ* 2.65X (n = 5–6, p<1x10^-3^) in the imaginal disc (Fig 2C, 2D vs. 2A, 2B) without affecting NS1 protein levels (Fig 2M). This NS1 mutant carried an alanine to valine substitution at position 122 (A122V)—a highly conserved residue mapping to the surface of the ED (Fig 2N). (Note: NS1(Vn) lacks 5 amino acids after position 79 that are present in almost all other strains. So, in order to remain consistent with the majority of NS1 variants, the NS1(Vn) amino acids were numbered with respect to their alignment with other NS1 sequences, effectively inserting blank sequence into the 5 amino acid deletion.) Substitutions of other, larger amino acids at position 122 also greatly reduced NS1 activity indicating that a key functional feature of the alanine residue is likely to be the small size of its side chain (see S1 Text and Table A in S1 Text for further structural requirements of this residue).

### Hh modulation via NS1 varies significantly between viral strains

**Fig 3:**
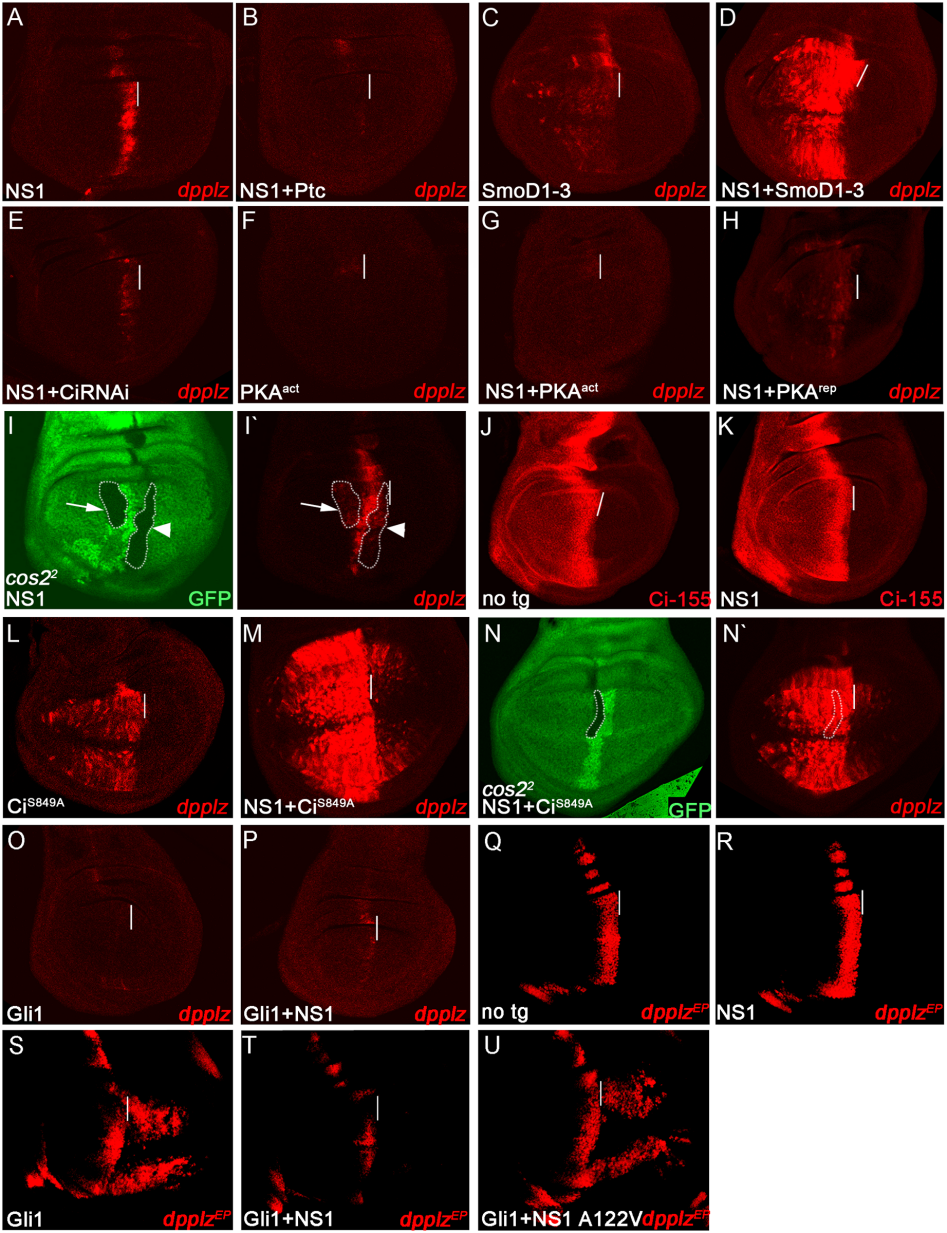
NS1 alters the activity of *Drosophila* Ci and mammalian Gli1. Wing discs ubiquitously expressing the indicated UAS-transgenes driven by 71B-GAL4 were stained for *dpp-lacZ* (A-H, I`,L,M,N`,O,P), Ci-155 (J,K), or *dpp-lacZ*^*EP*^ (Q-U) expression. NS1 activation of *dpp-lacZ* expression (A) was suppressed by co-expression with the inhibitory Ptc co-receptor (B) and potentiated by ubiquitous expression the activated SmoD1-3 co-receptor (C,D). Co-expression of NS1 with a Ci RNAi construct blocked NS1 activation of *dpp-lacZ* expression (E). Ubiquitous expression of PKA^act^ (F) which greatly reduces levels of Ci-155 by promoting its proteolysis and blocks *dpp-lacZ* expression also blocks the ability of NS1 to activate *dpp-lacZ* expression (G). Co-expression of NS1 with the inhibitory regulatory subunit of PKA (PKA^rep^) (H), or eliminating function *cos2* in mutant clones (lacking GFP and outlined in I and I`) prevents NS1 from activating high levels of *dpp-lacZ* expression (I`) in anterior (arrow) and A/P border cells (arrowhead). Ci-155 protein levels were similar in no transgene (J) and NS1-expressing (K) discs. NS1 enhanced Ci^S849A^-mediated ectopic expression of *dpp-lacZ* (L vs. M). *cos2* mutant clones (lacking GFP and outlined in N and N`) expressing activated Ci^S849A^ do not reduce NS1-dependent induction of *dpp-lacZ* expression (N`). Ubiquitous expression of mammalian Gli-1 blocked both endogenous (O) and NS1 dependent (P vs. A) expression of *dpp-lacZ*. NS1-dependent activation of *dpp-lacZ*^*EP*^ expression at the A/P border (R vs. Q) was also disrupted by co-expression with Gli1 (T vs. R). Similarly, NS1 disrupted ectopic posterior activation of *dpp-lacZ*^*EP*^ caused by ubiquitous expression of Gli1 (T vs. S). In contrast, Gli1-dependent activation of *dpp-lacZ*^*EP*^ was not disrupted when co-expressed with NS1 A122V (U vs. S).

Because different strains of influenza can vary dramatically in virulence, we examined the Hh modulating activity of NS1 from several viral strains including the standard seasonal Udorn virus, NS1(Ud), the more recently emerged swine flu NS1(Sw), and the murine adapted PR8 virus, NS1(PR8). NS1 transgenes from these strains were expressed with a strong wing specific driver so that even low levels of Hh inducing activity could be detected. NS1(Ud) produced wing phenotypes similar to those of NS1(Vn) (Fig 2E and 2F), while NS1(Sw) was 2.24X weaker as judged by relative *dpp-lacZ* expression at the A/P border of the wing imaginal disc (Fig 3G and 3H, n = 4, p<3.8x10^-3^). In contrast, NS1(PR8) was 7.9X stronger than NS1(Vn) (Fig 3I and 3J, n = 6, p<1.4x10^-3^). However, this greater activity of NS1(PR8) was also reduced 3.53X (n = 6, p<, p<6.4x10^-3^) by the A122V mutation (Fig 2K and 2L).

Since A122 is highly conserved between strains (not limited to just those we tested), we generated mutations in other surface amino acids close to A122 that differed between high (PR8) and low (Sw) activity forms of NS1 to identify residues that may contribute to NS1 activity (Fig 2N and Table A in S1 Text). These experiments revealed that no individual or tested combinations of residues contributed significantly to the variable activity between strains, suggesting that multiple amino acids, possibly encompassing different regions of the protein, contribute incrementally to the differential Hh-modulating activities of NS1.

### Highly active NS1(PR8) affects a broader range of Hh targets than NS1(Vn)

As described above, among the viral strains tested, NS1(PR8) was the most active in enhancing *dpp-lacZ* expression (Fig 2J). In addition, NS1(PR8) altered expression of Hh target genes that appeared unaffected by NS1(Vn) (e.g., Col, Ptc, and a narrow anterior En domain) (S1C, S1G and S1K Fig). These NS1(PR8)-specific effects included increased (e.g., *dpp-lacZ*: Fig 2J, Ptc: S1G Fig) as well as decreased (e.g., Col and En: S1C and S1K Fig) target gene expression. The differences in activity between NS1(PR8) and NS1(Vn) notwithstanding, incorporation of the A122V mutation into NS1(PR8) reversed its effects on all Hh target genes, indicating that this single amino acid is critical for both the positive and negative effects of NS1 across strains (Fig 2K and 2L and S1D, S1H and S1L Fig).

### NS1 alters the activity of Ci/Gli1

We next examined the effect of NS1 on Hh pathway components. Overexpressing the Ptc receptor, which sequesters Hh and blocks signaling by preventing it from diffusing into the anterior compartment [19,25], abrogated the ability of NS1(Vn) to upregulate *dpp-lacZ* expression (Fig 3A and 3B). Conversely, ectopic activation of Hh signaling in the anterior compartment, induced by ubiquitously expressing a constitutively active phosophomimetic form of Smoothened, potentiated NS1(Vn) activity throughout this region (Fig 3C and 3D). These results indicate that NS1(Vn) activity is dependent upon Hh signaling. To determine whether NS1 requires the presence of the full-length form of the transcriptional effector Ci (Ci-155), we reduced Ci-155 levels either by RNA-interference (Fig 3E), or by promoting proteolysis of Ci-155 to the Ci-75 repressor form by expressing the active catalytic subunit of PKA (PKA^act^) (Fig 3F and 3G) or Cos-2 (S4A and S4B Fig). In all cases, NS1(Vn) activity was abolished, demonstrating a requirement for Ci-155 in this process.

Full length Ci-155 requires additional modification(s) from the Hh signaling pathway for full activity (Ci^act^) [26]. To determine whether NS1(Vn) required such Ci activation, we co-expressed it with the inhibitory regulatory subunit of PKA (PKA^rep^, Fig 3H) or in clones of cells lacking function of Cos-2 (Fig 3I and 3I’), both of which block Ci phosphorylation/degradation and thereby stabilize Ci-155. Under these conditions, however, Hh signaling is also disabled due to required positive roles of both PKA and Cos-2 in transducing the Hh signal [27]. In these experiments, despite the presence of abundant Ci-155, NS1(Vn) was unable to fully activate *dpp-lacZ* expression. Thus, Ci-155 is required, but not sufficient, for NS1(Vn) activity and additional positive input from Hh signaling is essential for its full effect (see S1 Text and S4 Fig for further analysis). Consistent with NS1 affecting the specific activity of Ci rather than its expression or stability, levels and expression pattern of full-length Ci were unaltered by NS1(Vn) (Fig 3J and 3K) nor by the stronger variant, NS1(PR8) (S1M–S1P Fig).

In further support of NS1 acting in conjunction with Ci^act^, co-expression of NS1(Vn) with a non-cleavable variant of Ci-155, Ci^S849A^ [28], which is active independently of Hh signaling, strongly enhanced *dpp-lacZ* expression throughout the disc (Fig 3L and 3M). This effect of NS1 extended well beyond the domain of Hh signaling, and did so even in the absence of *cos-2* function (Fig 3N and 3N’). We conclude that NS1 interacts with fully-activated Ci^act^, which is normally present along the A/P border where Hh signaling is high.

We next examined whether Gli1, the mammalian transcriptional activator homologous to Ci [27,29], would similarly mediate a response to NS1(Vn). Unlike endogenous *ci* which is transcriptionally repressed in the posterior compartment of wing discs [30], both the mammalian and *Drosophila* effectors inserted into the *Drosophila* genome on UAS-transgenes can be expressed throughout the disc and subsequently activated in posterior and A/P border cells where the Hh ligand is abundant [31,32]. When we expressed Gli1 in *Drosophila* wing discs, we observed that it exhibited an opposing activity to that of Ci, as it nearly abolished the low basal level of expression of the synthetic *dpp-lacZ* reporter (Figs 3O vs. 1E) and also, unexpectedly, blocked the ability of NS1(Vn) to augment its expression (Fig 3P vs. 3A). Similarly, Gli1 prevented NS1(Vn) from fully activating expression of the more strongly-expressed *dpp*-*lacZ*^*EP*^ enhancer trap reporter along the A/P border (Fig 3T vs. 3Q and 3R). [30]. Likewise, Gli1 activity in posterior and A/P border cells was significantly impeded upon co-expression with NS1 (Fig 3T vs. 3S). Taken together, these results demonstrate an interaction between NS1 and Gli1. Futhermore, the A122V mutation eliminated this interaction such that NS1(Vn)-A122V could no longer prevent Gli1-mediated activation of *dpp-lacZ*^*EP*^ expression (Fig 3U vs. 3T). This indicates that the A122 residue is critical for interacting with the Hh pathway signaling effector across species.

NS1 was also found to regulate Notch (N) signaling in wing discs (see S1 Text and S4 Fig for further analysis). Commensurate with NS1 altering the activity of the transcriptional effector of the Hh signaling pathway, an interaction was also detected between NS1 and the transcriptional effector of the Notch (N) signaling pathway, N-ICD, which could also be relieved by the A122V mutation (S1 Text and S4 Fig). Similar to Hh signaling, Notch (N) signaling is also involved in tissue repair and immunity [12,33], suggesting that this novel binding domain of NS1 may be involved in various types of cell non-autonomous tasks that regulate the broader environment of the tissue during infection.

### NS1 interacts directly with Ci

**Fig 4:**
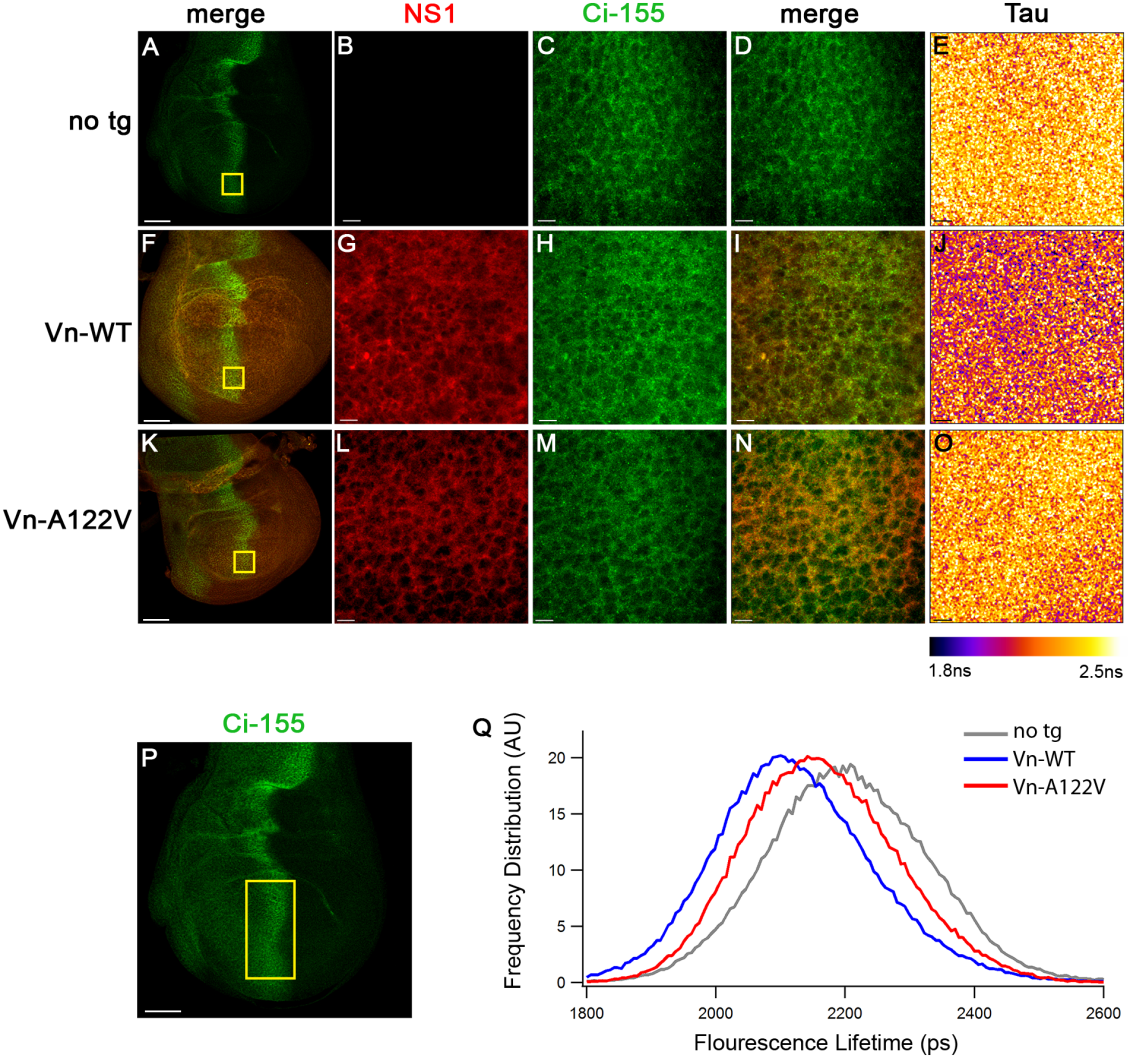
In situ evidence that NS1 interacts directly with Ci in *Drosophila* wing imaginal discs. (A-L) Wing discs with no transgene (no tg) or those ubiquitously expressing NS1(Vn)-WT or NS1(Vn)-A122V from a MS1096 wing-GAL4 driver were co-stained with Ci-155 and NS1 primaries and Alexa-488 (green) and Alexa-555 (red) secondaries, respectively. (B,C,D,G,H,I,L,M,N) High resolution images of cells at the A/P border show colocalization in staining patterns between NS1 and Ci-155 (yellow boxes in A, F, and K show the enlarged regions). (E,J,O) FRET-FLIM analysis in the selected ROIs show a spatially-resolved color table corresponding to fluorescence lifetime values (Tau) of Alexa-488 from 1.8ns to 2.5ns. NS1-WT (J) produces, on average, lower lifetime values of Alexa 488 compared to discs with no transgene (E) or those expressing the NS1-A122V transgene (O). The entire A/P border within the wing pouch (demarcated in P), was used to generate a plot of fluorescence lifetime values of Alexa 488 versus frequency of distribution (Q). The frequency of distribution (y-axis) is represented in arbitrary units (AU) and the fluoresence lifetime (x-axis) is represented in picoseconds (ps). The plot shows an average of 5 wing discs per group. Scale bars in A,F,K, and P represent 50um. Scale bars in B-E, G-J, and L-O represent 7um.

Consistent with the strong genetic interactions observed between NS1 and Ci, we found that full-length Ci and NS1 colocalize in A/P border cells of NS1-expressing discs, although no obvious differences in the staining patterns were observed between the wild type and mutant NS1 proteins (Fig 4F–4I and 4K–4N). In order to determine whether NS1 directly interacts with Ci in this functionally relevant region, wing imaginal discs were incubated with Ci-155 and NS1 antibodies, processed for immunofluorescence, and examined by fluorescence resonance energy transfer-fluorescence lifetime imaging (FRET-FLIM) analysis over a zone encompassing the A/P organizer (Fig 4E, 4J, 4O, and 4Q). Discs expressing NS1(Vn)-WT displayed decreased lifetime values (Tau) of the fluorescent secondary antibody bound to Ci-155 at the A/P border (1.9 to 2.2 ns) compared to discs with no transgene (2.1 to 2.4 ns), representing a range of 10–15% FRET efficiency [34] and, thus a direct interaction between NS1 and Ci-155 (Fig 4J vs. 4E and 4Q). Furthermore, expression of NS1(Vn)-A122V showed a significant reduction in this interaction, represented by lifetime values between 2.0 to 2.3 ns, resulting in 2–5% FRET efficiency and a 50% reversal in comparison to NS1(Vn)-WT expressing discs (Fig 4O vs. 4J and 4Q). These data provide strong evidence for NS1 interacting directly with Ci-155 along the A/P border and that mutation of A122 significantly impedes this interaction, implicating this residue in selective Ci binding.

### NS1 activity in mammalian cells

**Fig 5:**
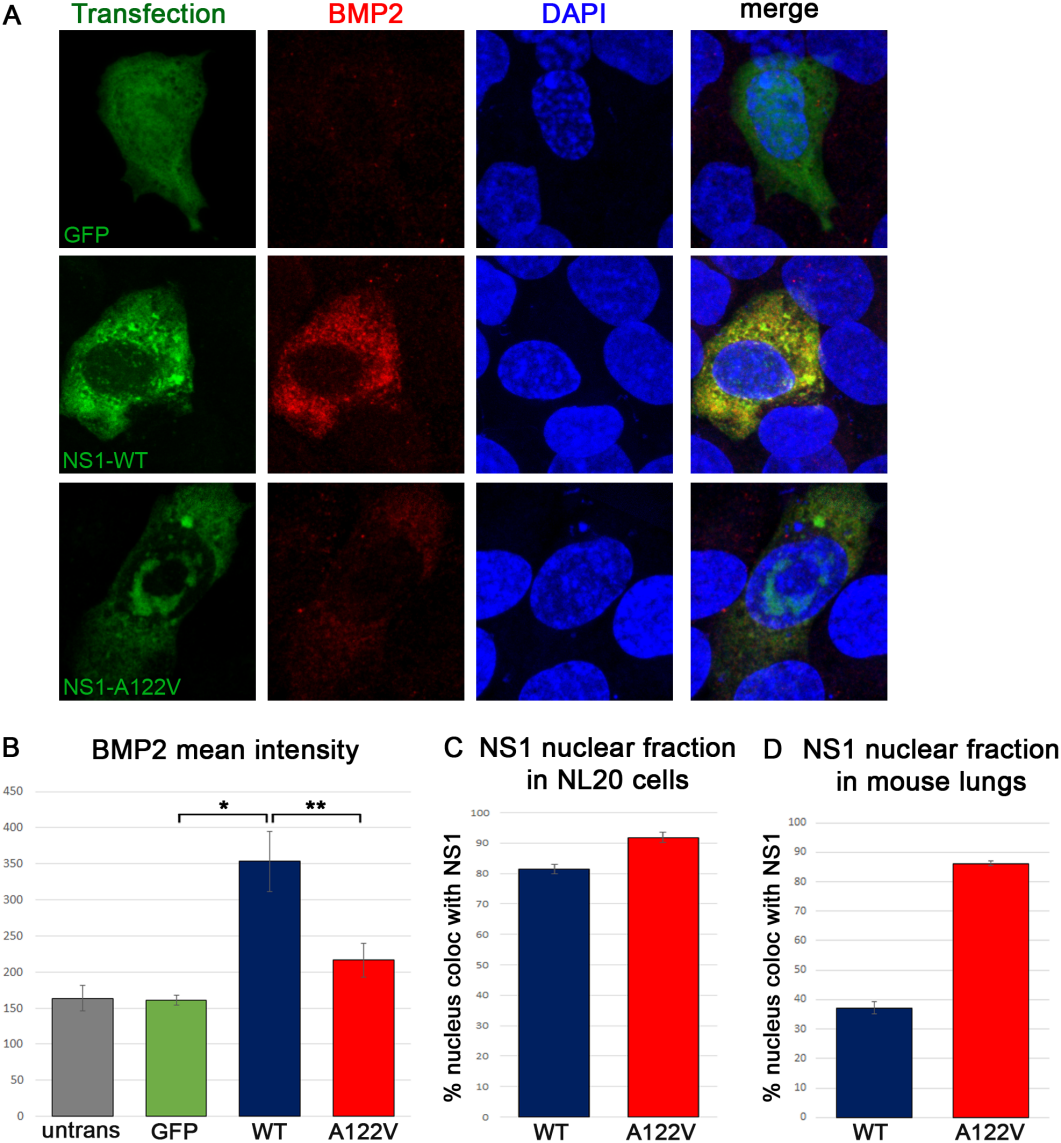
NS1 induces BMP2 expression in human lung cells. (A) NL20 cells transfected with plasmids expressing GFP (top row), NS1-WT (middle row) or NS1-A122V (bottom row) were stained with anti-NS1 (green) and anti-BMP2 (red) antibodies. Cells transfected with GFP (top row) were stained with anti GFP and anti-BMP2 antibodies. DAPI (blue) marks the nucleus. (B) The average mean intensity of BMP2 was quantified from 17–20 cells per group; *p = 3.04x10^-5^, **p = 9.5x10^-3^). (C) The fraction of nucleus colocalized with NS1 was quantified in NL20 cells (17–20 cells per group; p = 2.3x10^-5^). (D) The fraction of nucleus colocalized with NS1 (Fig 6A and 6C) was quantified from 10 regions of 1 infected lung per group (p = 3x10^-14^).

We next tested whether NS1 also exerted Hh-modulating activity in mammalian cells. Plasmids containing NS1(PR8)-WT-, NS1(PR8)-A122V-, or GFP-encoding sequences were transfected into the human lung epithelial cell line, NL20, and analyzed for expression of the Hh target gene BMP2, the mammalian homologue of *Drosophila dpp*. Compared to surrounding cells or cells expressing a GFP-expressing control transgene, cells expressing NS1(PR8)-WT proportionally induced BMP2 protein expression, as observed in flies (Fig 5A, 5B and S6A Fig). NS1(PR8)-A122V protein levels appeared generally lower than NS1-WT in NL20 cells (S6B Fig). Nonetheless, when cells with the highest levels of NS1(PR8)-A122V (A122V-high in S6B Fig) were compared to those expressing NS1(PR8)-WT, a clear reduction in BMP2 induction was still observed in the mutant-expressing cells (Fig 5A and 5B). These findings indicate that the Hh-modulating activity of NS1 is conserved between flies and humans, and moreover that this activity depends on the same A122 residue of NS1.

Interestingly, in NL20 cells (Fig 5A and 5C) as well as in mouse lungs infected with the PR8 strain of influenza virus (Fig 5D), we observed a higher proportion of NS1(PR8)-A122V in the nucleus as compared to NS1(PR8)-WT. In addition, mutant NS1 often formed nuclear accumulations or aggregates that were not observed with the wild type protein (Fig 5A). This supports a model in which the A122V mutation alters the specific activity of NS1 and/or its distribution and proximity to host binding partners.

### Infected mice display altered Hh target gene expression

We next tested whether Hh signaling was altered by influenza in a murine infection model, and whether the A122V mutation of NS1 had any effect on such activity. We used the PR8 virus for these experiments since NS1(PR8) is highly active for this function of NS1 and because PR8 virulence is independent of CPSF30 binding by NS1, a requirement for suppression of cellular mRNA maturation in other strains [35,36]. This latter fact was important since the A122V mutation in NS1(Ud), presumably due to its proximity to the CPSF30 binding site at M106, reduced this binding interaction *in vitro* (S7 Fig). Nonetheless, mutations of M106 or G184 which selectively eliminate CPSF30 binding did not alter NS1(Vn) activity in *Drosophila* (Table A in S1 Text), implying that CPSF30 binding is not involved in Hh signaling modulation by NS1.

**Fig 6:**
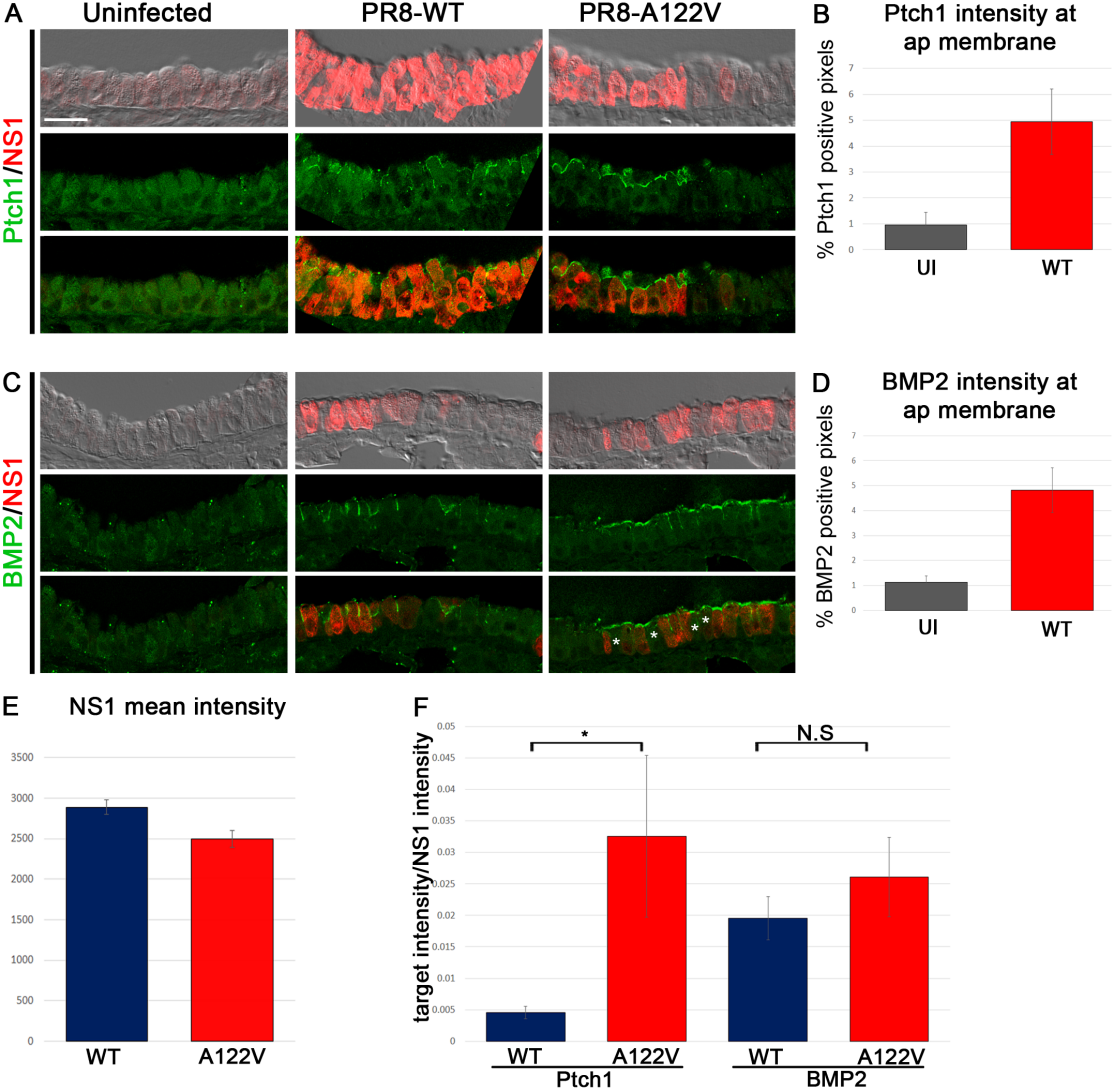
Hh target gene expression in infected mouse lungs. (A,C) Uninfected, PR8-WT, or PR8-A122V infected mouse lungs were stained 2 days post infection with anti-NS1 (red in A and C), anti-Ptch1 (green in A), and anti-BMP2 (green in C) antibodies Asterisks in C denote neighboring uninfected cells that show an upregulation of BMP2. (B, D) Intensities of Ptch1 (B) and BMP2 (D) were quantified in uninfected and PR8-WT infected lungs. In B, 11–20 confocal fields from two mice per group were quantified (p = 0.03). In D, 12–20 confocal fields from two mice per group were quantified (p = 4.1x10^-3^). (E) The mean intensity of NS1 was quantified from 25 confocal fields from two mice per group (p = 5.6x10^-3^). (F) The relative intensities of Ptch1 and BMP2 were normalized to NS1 intensity from 20–23 confocal fields from two mice per group (*p = 0.04).

Sibling mice were infected with either the PR8-WT or PR8-A122V virus and lung airway epithelium were analyzed for Hh target gene expression 2 days post-infection. Compared to uninfected lungs, the canonical Hh-target genes, Ptch1 (Fig 6A and 6B), and BMP2 (Fig 6C and 6D) were both strongly up-regulated in infected lungs in a cell-autonomous fashion at the apical plasma membrane, a ciliated region of the cell where Hh components assemble [37]. Local non-autonomous effects were also occasionally observed in cells immediately adjacent to infected cells suggesting that virally infected cells exert effects on surrounding, ostensibly healthy, cells (asterisks in Fig 6C).

Similar to NL20 cells, NS1 expressed from the PR8-A122V mutant virus was found at slightly but statistically significantly lower levels compared to NS1 expressed from the WT virus (Fig 6E). Despite the lower levels, and in contrast to the diminished activity of NS1-A122V when expressed in flies and in transfected cells, the mutated virus induced higher expression of the Hh target Ptch1 compared to PR8-WT (Fig 6A and 6F). However, PR8-A122V did not discernibly alter the expression of BMP2 compared to PR8-WT. Taken together, NS1-A122V has altered activity compared to NS1-WT in flies, human cells, and *in vivo* in mice. Potential reasons for differences between the activity of NS1 assayed in isolation versus in the context of viral infection *in vivo* are considered below in the discussion section.

### PR8-A122V is more pathogenic than PR8-WT

**Fig 7:**
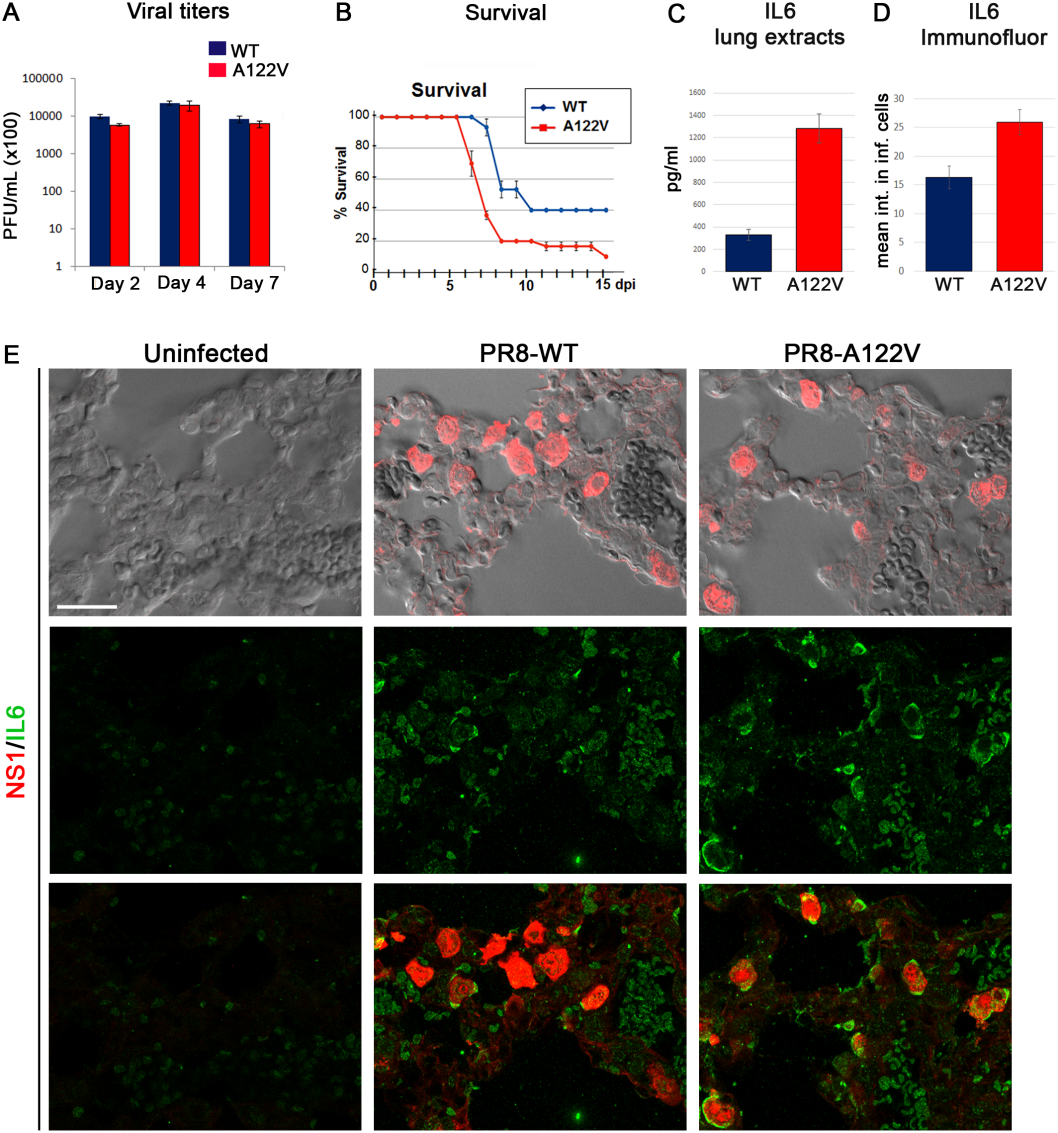
IL6 is expressed cell-autonomously in infected cells in mouse tissue. (A) Viral titers were measured for mouse lungs infected with the PR8-WT (blue) and PR8-A122V (red) viruses at 2, 4, and 7 days post infection. (B) 25 mice per group compiled from three independent experiments were infected with either the PR8-WT (blue) or PR8-A122V viruses (red) at 1x10^4^ pfu and monitored daily for survival (p = 0.0001). (C) IL6 levels in lung extracts were higher in PR8-A122V infected lungs compared to PR8-WT (n = 4–5, p = 0.005). (D) PR8-A122V infected cells in mouse lung tissue had higher intensity values of IL6 compared to PR8-WT infected cells. 12–20 confocal fields from two-three mice per group were quantified (p = 0.005). (E) Uninfected, PR8-WT, or PR8-A122V infected mouse lungs were stained 2 days post infection with anti-NS1 (red) and anti-IL6 (green) antibodies.

Having observed an effect of PR8-A122V in the context of *in vivo* infection in mice, we next examined its physiological effects. Both the PR8-WT and PR8-A122V viruses established comparable levels of infection as assessed by viral titers in lungs (Fig 7A) and the range of tissues infected in mice at either two, four, or seven days post infection (influenza was below the limit of detection in the brain, spleen, and liver)), and displayed similar temporal regulation of viral genes in cell culture (see S1 Text and S8A Fig). However, the PR8-A122V virus significantly hastened lethality of mice compared to the parental PR8 (PR8-WT) strain in three independent experiments (Fig 7B) and produced greater signs of morbidity by 3 days post-infection (e.g., reduced mobility, hunched posture, labored breathing, and pilo-erectus, although insignificant change in weight loss–S8B Fig), indicating that the mutant virus is considerably more pathogenic than the parental strain. This observation suggests that lower levels of Hh target gene induction by NS1-WT during infection *in vivo* may serve a protective function for the host whereas unrestrained signaling, as occurs during infection with PR8-A122V, may be more detrimental.

To determine whether the increased pathogenicity might be linked, at least partially, to the induction of cytokine storms (an uncontrolled positive feed-back loop between immune cells and cytokine production thought to have caused many fatalities during the past influenza pandemics) cytokine levels were measured in infected lung extracts [38–40]. Indeed, levels of the proinflammatory cytokines IL6 (Fig 7C) and CXCL-10 (S9 Fig) were significantly higher in extracts (lavages) from lungs infected with PR8-A122V compared to PR8-WT viruses, while other factors such as TNF-α and IL-1α were not significantly altered (S9 Fig).

It has recently been shown that Hh signaling can directly modulate the immune response by activating expression of cytokines, such as IL6 [13,41,42]. Thus, we sought to determine whether the upregulation of IL6 observed in infected lungs (Fig 7C) might be a consequence of the direct activation of Hh signaling by NS1, or rather derives from a general immune response. We detected both a cell-autonomous effect of influenza inducing an increase in IL6 expression in infected cells, as well as a general non-autonomous increase in mouse lung tissue (Fig 7E). Furthermore, IL6 expression was higher in PR8-A122V infected cells compared to PR8-WT (Fig 7D and 7E). Thus, the hastened lethality occurring in mutant-virus infected animals may be due, in part, to a direct interaction between NS1 and Hh signaling causing unrestrained expression of some cytokines.

## Discussion

### Identification of novel effects of NS1 on Hh signaling

In this study, we identified a novel effect of the influenza virulence factor NS1 in modulating Hh signaling. This Hh altering activity was initially identified using the *Drosophila* wing as a discovery model system. Then, we demonstrated a conservation of this activity in vertebrate systems such as transfected cultured human lung cells, as well as in influenza-infected mice. Detailed mechanistic analysis in *Drosophila* established that NS1 acts at the level of the transcriptional effector, Ci/Gli1, and FRET data strongly suggest that this interaction is direct. The effect of NS1 on Hh target gene expression in *Drosophila* was either positive or negative depending on the particular target gene, NS1 strain, and co-expression of *Drosophila* Ci or vertebrate Gli partner. The positive versus negative effects of NS1 on particular target genes may reflect promoter-specific interactions in which the presence of NS1 can either stabilize or disrupt critical interactions between transcription factors, co-factors, and other transcriptional machinery.

Importantly, the single amino acid substitution, A122V, which was recovered by a forward genetic screen in *Drosophila*, dramatically altered most NS1 responses in flies, human lung epithelial cells, and airways of infected mice. Furthermore, mutations abrogating interactions with several known host effectors involved in viral replication or suppression of the interferon response had little, if any, effect on the Hh signaling function of NS1 assayed in flies. These observations suggest that a novel, highly specific interaction surface of NS1 mediates these modulatory effects on Hh signaling.

The above results strongly implicate NS1 as a modulator of Hh signaling, however, differences in its activity could be observed depending on the cellular context. Most notably, NS1-A122V, when expressed independently of other viral proteins (i.e., in flies and cultured human cells) had reduced activity compared to NS1-WT. However, this same mutation resulted in a more pronounced inflammatory response *in vivo* in mice where we observed higher expression of some Hh targets, and greater virulence when assayed in the context of viral infection (i.e., in PR8 infected mouse lungs) suggesting that other viral factors contribute to determining the net effect of the NS1-A122V mutation on the Hh response. One model that could account for these differences involves specific interactions between NS1 and nuclear factors. We have shown that NS1-A122V localizes more to the nucleus than NS1-WT. Thus, during viral infection, the spectrum of interactions between NS1, viral factors, host defense components, and Hh targets, occurring specifically in the nucleus may be altered preferentially by the A122V mutation. Increased nuclear levels of NS1-A122V coupled with a reduced interaction with Ci/Gli1 (as was detected in flies) might favor alternative NS1-effector interactions that lead to a more pronounced inflammatory state. Viral factors that shuttle between the nucleus and cytoplasm, such as the matrix protein (M1), nucleoprotein (NP), and nuclear export protein (NEP), may be key in mediating these interactions [43].

### The Hh signaling pathway as a target of influenza

Hh signaling is a plausible target for manipulation by pathogens, as this pathway plays an integral role in cell survival and proliferation [10,11,16]. Indeed, the Hh target gene Ptch1 was identified in an independent study as a critical host gene involved in influenza infection [44]. Therefore, our study provides a mechanistic interpretation for this observation, and reinforces the idea that controlling Hh signaling during influenza infection may favor viral dissemination. One way in which constraining Hh signaling by NS1 may be beneficial to the virus is by limiting damage to the lung in order to preserve the viral habitat. Hh signaling has been shown to be induced in the lung by damage caused by chemical agents and many types of ailments [45–48]. Furthermore, the Hh receptor, Ptc, is expressed in infiltrating and circulating lymphocytes suggesting that immune cells are primed to respond to the Hh ligand secreted from the inflamed area [45]. These studies indicate a key role for Hh signaling in repairing damaged lung tissue by remodeling the epithelium through, or in conjunction, with activated immune cells. Similarly, in fruit flies, Hh signaling has been shown to play a critical role in remodeling the epithelial barrier in response to pathogen infection [15]. However, too much remodeling by overactive signaling has also been shown to lead to the formation of detrimental fibrotic tissue [49]. In the case of influenza infection, Hh signaling may be activated indirectly to help repair the damaged lung epithelium. However, activation in an uncontrolled manner, such as that induced by PR8-A122V, may result in more fibrotic tissue damage. Thus, controlling the Hh response may help the host avoid such deleterious effects to the infected tissue and improve viral survival as well as dissemination.

Unrestrained Hh signaling could also be detrimental to the host by excessively activating expression of cytokines, such as IL6 [13,41,42]. Indeed, we found that influenza infection strongly induced CXCL-10 and IL6 expression, the latter of which was partially enabled by a direct interaction between Hh signaling and NS1. As IL6 was present at significantly higher levels in animals infected with PR8-A122V compared to PR8-WT, we speculate that the hastened lethality by the mutant virus may be caused, at least partially, by a direct induction of cytokine storms by NS1. Other cytokines regulated by the Hh pathway such as IL-8, Mcp-1, and M-csf could also contribute to this phenotype as well [41]. Furthermore, the fact that no mutation at position 122 of NS1 (or the analogous position in other viral strains) has been identified previously in any influenza strain, may reflect the critical role of NS1 in dampening the level of cytokine activation, resulting in optimized host survival and/or viral spread. Interestingly, our initial screen in *Drosophila* revealed that NS1 from Swine flu (NS1(Sw)) behaved similarly to the A122V mutant of other strains despite the lack of such a mutation. This may reflect an inability of this particular NS1 protein to regulate Hh signaling and restrain cytokine induction which may be directly linked to the augmented mortality rate of this virus observed in several animal models [50–53]. Indeed, many pro-inflammatory cytokines, including IL6, were detected at higher levels in mouse and cynomolgus macaque lungs infected with this particular strain [51]. NS1 proteins from other influenza stains may act similarly to NS1(Sw), thus initial screening in *Drosophila* may prove a useful method of defining the virulence of emerging viral strains.

### Implications for potential new therapeutic strategies to treat influenza infection

There is a precedent for other viruses interacting with the Hh pathway, such as Epstein Barr Virus [54], and Hepatitis B and C [55]. At least in the case of Hepatitis B and C, treatment of cells and tissue with a potent Hh antagonist appears to significantly limit viral outputs [56,57]. Additionally, blocking Hh signaling with the conventional antagonist, Cyclopamine, can often limit the extent of inflammation and fibrosis in other types of distressed tissue as well [58–61]. Therefore, further restricting Hh signaling with small molecule antagonists, such as Vismodegib (GDC-0449), which has been US-FDA approved as a treatment for basal cell carcinoma, represents a promising avenue to explore as a treatment for influenza [62]. In contrast to the currently available therapies such as the seasonal vaccines and antivirals that target strain-specific and rapidly-mutating viral proteins, treatments that target highly-conserved viral host targets may ultimately provide superior and continual protection across a broader spectrum of viral strains.

In conclusion, our study demonstrates a novel activity for NS1 in modulating Hh signaling during influenza infection which elicits, to some extent, protection to the host. This effect is dependent upon a direct interaction with the Ci/Gli transcription factor, but the precise mechanism by which NS1 exerts it effects on the Hh pathway during infection remains to be addressed. Potential new therapies involving Hh inhibitory compounds could derive from these findings and demand further investigation.

## Materials and methods

### Ethics statement

Animal care and breeding are performed in the AAALAC accredited vivarium (Vertebrate Animal Assurance No. A3194-01) at The Scripps Research Institute. For mouse experimentation and euthanasia, the AAALAC and NIH guidelines were followed and approval was given by the TSRI animal committee. Mice were euthanized when moribund to prevent undue suffering, and were sacrificed when they showed a failure of locomotion/activity and stayed huddled in a corner of the cage. These mice were unable to eat or drink and showed respiratory difficulties. Pilot studies showed that such mice would die within 24 hours.

### Immunohistochemistry of fly wing imaginal discs

Generation of mitotic clones and immunohistochemistry was performed as previously described in [28]. All antibodies used are listed in S1 Text.

### Immunohistochemistry of mouse lungs

Lungs were harvested at 2 dpi and placed in PBS-buffered formalin, blocked in paraffin, 10-μm tissue sections were cut, and placed on glass slides. Lung sections where deparaffinized in Xylenes (3X 10’), and washed in 100% Ethanol (2X 5’). After progressive rehydration (95% Ethanol 2X 5’, 70% Ethanol 1X 5’), slides were rinsed 3 times in 1X PBS. Antigen unmasking was performed using a reagent from Vector (H-3300, 2.5ml in 250ml H2O), and microwaving 2X for 5 minutes, followed by a 20 minute cool down, and 2 PBS1X rinses at RT. Lungs sections were outlined using an Elite Mini PAP Pen (Diagnostic BioSystems K 042), and incubated in blocking buffer (1XPBS with 1% BSA) for 30’. Incubation with primary antibodies primary antibodies was performed overnight at 4°C in a humid chamber followed by 3X PBS rinses. Secondary incubation was performed in blocking buffer followed by 3X rinses in PBS. Slides were mounted using VectaMount AQ (Vector, H 5501).

### Maintenance, transfection, and immunohistochemistry of NL20 cells

NL20 human lung epithelial cells (ATCC) were maintained in a 5% CO2 atmosphere at 37°C in Ham's F12 medium (Gibco) supplemented with 1.5 g/L sodium bicarbonate, 2.7 g/L glucose, 2.0 mM L-glutamine, 0.1 mM non-essential amino acids (Lonza, 13–1146), 0.005 mg/ml insulin (Sigma-Aldrich), 10 ng/ml epidermal growth factor (Corning), 0.001 mg/ml transferrin (Lonza), 500 ng/ml hydrocortisone (Sigma-Aldrich) and 4% fetal bovine serum. Transfections were performed on monolayers grown on Collagen-coated (Gibco, A10483-01) coverslips and transfected for 24 hours with 0.5 μg of the indicated plasmids using X-tremeGENE 9 DNA Transfection Reagent (Roche), according to the manufacturer’s instructions. Cells were washed with PBS, fixed with 4% formaldehyde and stained with primary antibodies. After incubation with Alexa fluor-conjugated secondary antibodies in blocking buffer for 1 hour at RT, cells were mounted with ProLong Diamond antifade mountant with DAPI (Molecular Probes) for microscopy analysis.

### Generation of wild type and recombinant influenza viruses

The influenza A/PR/8/34 (PR8; H1N1) wild type virus was rescued as previously described [63]. Alanine 122 in the PR8 NS1A protein was changed to valine using site-directed PCR mutagenesis of the PR8 NS gene, and the resulting DNA was cloned into a pol-lI plasmid, pHH21. This mutation does not change the sequence of the NS2 protein. Viruses were amplified as described previously [36]. All viral segments were sequenced in their entirety.

### Mouse infections

C57BL/6 mice were infected intra-tracheally with A/PR/8/34; H1N1 viruses at 10^4^ PFU and monitored daily for morbidity for 14 days. Alternatively, mice were anesthetized with isoflurane and lungs dissected. Cytokine production was measured from lung homogenates by ELISA assay using DuoSet kits from R&D systems (R&D, Minneapolis MN). All infected mice were housed in biocontainment at the animal facility of TSRI. Quantification of viral titers in mouse lungs is described in S1 Text.

Details of fly genetics, Western blotting, antibodies, cloning constructs and techniques, confocal and FRET-FLIM imaging, quantification, and statistics can be found in S1 Text.

## Supporting information

S1 Text Additional activities and in-depth analysis of NS1. (DOC)

S1 Fig NS1(Vn) versus NS1(PR8) on other Hh target genes. Wing imaginal discs with no transgene (no tg) or ubiquitously expressing the indicated transgenes were stained with antibodies recognizing Collier (Col) (A-D), Ptc (E-H), Engrailed (En) (I-L), and Ci-155 (M-P). The moderately active NS1(Vn) has little, if any, effect on expression Col (B vs. A), Ptc (F vs. E), or Hh-dependent anterior En expression which is present in late third instar wing discs (J vs. I), or full length Ci (N vs. M). The more strongly active NS1(PR8), however, results in reduced expression of Col (C vs. A), ectopic expression of Ptc (G vs. E), and reduced anterior expression of En (K vs. I). Each of these NS1(PR8) activities is greatly attenuated by the A122V mutation (D, H, L). Similar to NS1(Vn), NS1(PR8) does not appreciably alter Ci-155 levels (O,P). White lines in I-L denote the A/P border.
(TIF)

S2 Fig Secreted Dpp mediates the non-autonomous effect of NS1 on neighboring cells. Wing discs expressing the indicated transgenes with the ubiquitous 71B-GAL4 driver (A-F), or along the A/P border with the dpp-GAL4 driver (G, H), were assayed for the pattern of Dpp response by staining with a phospho-Smad1 antibody (pMad). Ubiquitous expression of a wild-type form of the Dpp type-II receptor subunit Thick veins (Tkv) restricts Dpp signaling to the A/P border (A). While pMAD staining is restricted to the A/P border, NS1 activity is not compromised (B). Expression of a dominant negative form of the Tkv receptor subunit eliminates both endogenous and NS1-activated pMAD staining (C, D). Ubiquitous expression of the potent secreted SogCR1 Dpp antagonist (Yu et al., 2000) also results in strong reduction of the NS1 response (E, F), while A/P border expression of SogCR down-regulates the response strongly in A/P border cells but seems to allow some signal to escape into adjacent regions which can be enhanced by NS1 (G, H).
(TIF)

S3 Fig NS1-R38A has reduced activity but can still modulate Hh signaling. (A) A wing with no transgene (no tg) with demarcated longitudinal veins L2-L5, M = margin. Wings expressing NS1(Vn)-R38A (C,E) have a reduced phenotype compared to wings expressing wild type NS1(Vn) (B,D) with either the moderate wing GAL4 driver, 71B (B,C) or the stronger driver, MS1096 (D,E). (F-L) Wing discs expressing the indicated transgene were assessed for NS1 activity by measuring *dpp-lacZ* expression (F,G,H,I,J,K,L). NS1 protein levels were also analyzed by staining discs with a Myc antibody directed towards the C-terminal tag of the transgene (F'.G',H',I'). NS1(Vn)-R38A appears to have lower protein levels (G' vs. F' and I' vs H') and less ability to activate *dpp-lacZ* expression when expressed with 71B-GAL4 (G vs. F). However, when expressed with the stronger driver, Apterous-GAL4 (Ap) which is expressed only in the dorsal compartment of the wing disc (green area in J'), NS1(Vn)-R38A is able to enhance *dpp-lacZ* expression in this domain (I) compared to discs expressing a GFP construct (J) and to similar levels as NS1(Vn) (H). NS1(Vn)-R38A is also able to enhance Ci^S849A^-mediated ectopic expression of *dpp-lacZ* (L vs. K).
(TIF)

S4 Fig Analysis of Hh pathway components required for NS1-dependent *dpp* activation. Reducing the levels of Ci-155 by ubiquitously expressing Cos-2 blocks endogenous *dpp-lacZ* expression (A), and also blocks the ability of NS1 to enhance *dpp-lacZ* expression (B). In *fused (fu*^*mH63*^*)* mutant discs, *dpp-lacZ* expression is broadened but not elevated (C). Expression of NS1 in a *fu* mutant background can augment *dpp-lacZ* expression throughout the expanded response zone (D), indicating that *fu* is not required for the *dpp*-activating function of NS1, although Fused is required for full activation of Ci-155. NS1(Vn) can enhance *mtv-lacZ* expression at the A/P border and activate expression throughout a broad domain in the anterior compartment (F vs. E). In clones of cells expressing a strong hypomorphic allele of *mtv (mtv*^*6*^, marked by the absence of GFP in G and outlined in G and G`), high NS1-dependent expression of *dpp-lacZ* is unaffected (G`). In discs expressing a hypomorphic allele of *kn* (*kn*^*1*^), expression of the *dpp-lacZ* reporter construct is not significantly altered (H), nor is the ability of NS1(Vn) to elevate expression of this reporter (I). Where present, transgenes were expressed with the ubiquitous 71B-GAL4 driver.
(TIF)

S5 Fig NS1 alters Notch target gene expression in a strain specific and A122V-dependent fashion. NS1 (Vn) reduces expression of Cut along the presumptive wing margin (B vs. A) and increases expression of *Gbe-lacZ*, a synthetic Notch reporter gene construct (E vs. D). Both of these effects of NS1 (Vn) are greatly reduced by the A122V mutation (C vs. B and F vs. E). The effect of NS1 (PR8) is stronger than NS1 (Vn) with regard to Cut repression (G vs. B) and *Gbe-lacZ* activation (I vs. E), and both of these effects are reduced by the A122V mutation (H vs. G and J vs. I, respectively). NS1(Vn) also blocked ectopic expression of Cut induced by ubiquitous expression of the activated N-ICD transcriptional effector (L vs. K).
(TIF)

S6 Fig BMP2 expression levels in human cells is proportional to NS1 expression levels. (A) NL20 cells transfected with the indicated NS1-expressing plasmids show an induction of BMP2 expression proportional to NS1 expression. (B) The mean intensity of NS1 was quantified from 17–20 cells per group (*p = 0.021).
(TIF)

S7 Fig The A122V mutation disrupts NS1(Ud) binding to CPSF30. GST or GST-NS1 fusion proteins were incubated with extracts of 293T cells transfected with an N-terminal fragment of CPSF30 containing four of its zinc finger binding domains and tagged C-terminally with a V5 epitope. Proteins immune precipitated with glutathione beads were visualized on Western blots with anti-V5 (top) and anti-GST (bottom) antibodies. The A122V mutation reduces NS1 binding to CPSF30 under these *in vitro* binding conditions.
(TIF)

S8 Fig The A122V mutation does not affect temporal regulation of viral proteins in infected tissue culture cells or affect weight loss in mice. (A) Temporal synthesis of two viral proteins, NP and M, was examined in A549 cells following a high-MOI single cycle infection with the PR8-WT and PR8-A122V viruses. No differences in the levels or kinetics of these markers were observed indicating that the A122V mutation does not alter temporal regulation of viral gene expression. (B) 10 infected mice per group were monitored for weight loss for 15 days. There was no significant difference in weight loss between PR8-WT and PR8-A122V infected animals.
(TIFF)

S9 Fig Cytokine levels in infected mouse lungs. CXCL-10, TNF-α, and IL-1α levels were measured from extracts of mouse lungs infected with PR8-WT and PR8-A122V. CXCL-10 was significantly increased in mutant infected lungs compared to the WT (p = 0.043), whereas TNF-α, and IL-1α were not appreciably changed. 4–5 lungs were analyzed per group.
(TIF)
