# Supplementary material for: Influenza NS1 directly modulates Hedgehog signaling during infection
Source: PLoS Pathog. 2017 Aug 24;13(8):e1006588. doi: 10.1371/journal.ppat.1006588 (PMC5587344; doi:10.1371/journal.ppat.1006588)
Supplement: S1 Text — (DOC) [file ppat.1006588.s001.doc]

**S1 Text. Additional activities and in-depth analysis of NS1**

**NS1 activates Dpp signaling cell non-autonomously by promoting higher expression of *dpp* at the A/P border in *Drosophila* wing imaginal discs**

One way to test for the non-autonomous effect of a factor that regulates BMP signaling is to determine whether it can alter the amount of free Dpp ligand diffusing into neighboring domains. Ubiquitous expression of the receptor, Tkv, inhibits Dpp diffusion and results in restricted activation of signaling in Dpp producing cells along the A/P border (Fig. S2 A). As expected, when NS1(Vn) was co-expressed with Tkv we found that it enhanced Dpp signaling selectively along the A/P border (Fig. S2 B). Furthermore, co-expression of NS1(Vn) with a dominant negative form of Tkv or with the antagonist Short Gastrulation (Sog), a secreted protein that sequesters Dpp away from the receptor, completely blocked NS1-induced Dpp signaling and thus phosphorylation of Mad (Fig. S2 C-F) [1,2] (our unpublished data). In contrast, NS1(Vn) retained the ability to enhance Dpp signaling in neighboring cells when Sog expression was restricted to the A/P border, a configuration that blocks Dpp activity locally, but allows (and possibly facilitates) Dpp diffusion (Fig. S2 G,H). From these observations, we conclude that NS1(Vn) increases long range Dpp signaling by increasing the level of *dpp* expression, and hence Dpp protein production, within Hh responding cells along the A/P border.

**The Hh modulating effect of NS1 is not mediated by known host effectors**

NS1 binds to several known host effectors via specific interaction surfaces. Mutations have been identified in NS1 that selectively abrogate specific interactions with dsRNA or with its various host target proteins (all of which have homologues or closely related genes in *Drosophila*) [3]. Host factors inhibited by binding to the NS1 ED include: protein kinase R (PKR), which halts cellular and viral protein synthesis; the 30 kDa subunit of cleavage and polyadenylation specificity factor (CPSF30) and poly(A)-binding protein II (PABII), which are required for mRNA maturation; and components of the nuclear export machinery (NXF1, p15, and Rae1) [3]. The NS1 ED can also bind to the p85β regulatory subunit of phosphoinositide 3-kinase (PI3K) and, in some strains, Crk/CrkL which may act to stimulate proliferation and reduce apoptosis in virally-infected cells [3]. In addition, residues spanning both the RBD and ED of NS1 have been implicated in temporal regulation of vRNA synthesis and selective translation of viral mRNAs [3].

We therefore asked whether the NS1-dependent induction of *dpp* expression was due to an interaction with any of these known host factors by mutating the residues essential for those interactions and expressing the mutated transgenes in flies (Table A). With only one exception (ds-RNA binding), none of these mutations altered *dpp* expression or pMAD staining. Mutations abolishing dsRNA-binding did reduce NS1 activity (Fig. S3 A-E, F,G) as well as protein levels in several lines (Fig. S3 F',G',H',I'). However, higher level of expression of such NS1 mutant transgenes had similar effects to the wild-type NS1 (Fig. S3 H,I) and were able to stimulate *dpp-lacZ* expression when co-expressed with activated Ci^S849A^(Fig. S3 K,L). We conclude that the Hh inducing activity of NS1(Vn) is not mediated by host effectors that bind to previously identified NS1 interaction surfaces.

**Table A. Relative NS1 activity in Drosophila wings and wing discs**

| **Known mutations** | ***dpp-lacZ* expression** | **Adults phenotype** | **Reason for change/expression** |
| --- | --- | --- | --- |
| **Vn WT** | 4 | 4 |  |
| **Vn WT+PolyA binding protein [3]** | 4 | 4 | NS1 is known to bind PolyA binding protein |
| **Vn WT+PI3K[3]** | 4 | 4 | NS1 is known to bind PI3K |
| **Vn and Ud R38A* [4]** | 1 | 1 | Eliminates dsRNA binding |
| **Vn G184R [5]** | 4 | 4 | Eliminates binding to CPSF30 |
| **Vn I123, M124A [6]** | 4 | 4 | Eliminates binding to PKR |
| **Vn P164, 167A [7]** | 4 | 4 | Eliminates binding to the p85β subunit of PI3K |
| **Vn P210A [8]** | 4 | 4 | Mutation of the SH3 domain, binding site to Crk family of proteins in some strains |
| **Vn Splice mutant** | 4 | 4 | Eliminates NEP fragment |
| **Vn A122V** | 1 | 1 | Identified in EMS screen |
| **Vn A122I** | 1 | 1 | Bulkier, hydrophobic residue at 122 |
| **Vn A122S** | 2.5 | 2.5 | Hydrophilic residue at 122 |
| **Vn L105W** | 3.5 | 2.5 | Bulkier, hydrophobic residue at 105, on protein surface near A122 |
| **Vn L105S** | 4 | 4 | Hydrophilic residue at 105, on protein surface near A122 |
| **Vn A122V, L105W** | 1 | 1 | Combine mutations at 105 and 122 |
| **Vn M106F** | 4 | 4 | Bulkier, hydrophobic residue at 106, on protein surface near A122. Also a CPSF30 binding site |
| **Vn M106S** | 4 | 4 | Hydrophilic residue at 106, on protein surface near A122 |
| **Vn M106I, F98S** | 4 | 4 | Changed to PR8 residues, on protein surface near A122 |
| **Vn D125E, D189G** | 4 | 4 | Changed to Sw residues, on protein surface near A122 |
| **PR8 WT** | 8 | No adults |  |
| **PR8 A122V** | 5 | 5 (escapers) | Verify A122V also reduces PR8 effect |
| **PR8 I106M** | 8 | No adults | Changed to Vn residues, on protein surface near A122 |
| **PR8 I106A** | 8 | 7 (escapers) | Changed to A as to avoid binding to CPSF30 |
| **PR8 I106M, A122V** | 5 | 5 (escapers) | Combined mutations at 106 and 122 |
| **PR8 I106, A122V** | 5 | 5 (escapers) | Combined mutations at 106 and 122 |
| **Sw WT** | 1 | 1 |  |
| **Sw R108K, I111V, V117I** | 1 | 1 | Changed to Vn residues, near A122 |
| **Sw R108K, I111V, V117I, E125D, G189D** | 1 | 1 | Changed to Vn residues, near A122 |

Table legend: Indicated constructs were expressed in fly wing imaginal discs and evaluated for enhancement of *dpp-lacZ* expression and adult wing phenotypes. Phenotypic strength is on a scale from 1-8 with 8 being the highest. The rationale for the experiment and citations, where available, are also listed. *See S1 text for further characterization of NS1 R38A mutant.

**Structural requirements of NS1 residue 122 that enable Hh modulating activity**

We explored structural features of the amino acid at position 122 essential for mediating the Hh modulating activity of NS1. A change from alanine to valine is rather subtle with the latter having only a slightly bulkier side group, yet remaining hydrophobic. To test whether the size of the side group was critical, we expressed NS1(Vn) transgenes in the wing with residues of different size and/or hydrophobicity incorporated into position 122. Isoleucine, a hydrophobic residue with an even bulkier side group than valine, gave a similar phenotype as A122V (Table A). In contrast, a transgene with serine at this position, which is bulky yet hydrophilic, did not greatly compromise NS1 function (Table A). These results suggest that a small hydrophobic residue at this position is the most important feature for mediating the Hh modulating activity of NS1 and that bulkier, hydrophobic residues (i.e. Val or Ile) may sterically block this binding interface.

**NS1 alters Notch signaling at the level of its transcriptional effector**

Notch signaling is required for a variety of binary cell fate decisions in many developmental contexts including specification of the dorsal-ventral compartment border in the *Drosophila* wing primordium [9]. Additionally, N signaling has also been implicated in several aspects of innate and adaptive immunity forming an important bridge between antigen presenting cells and T-cell activation circuits making it a desirable target for viral control [10].

When activated by the Delta or Serrate ligands, the Notch receptor undergoes a series of cleavage events leading to the generation of a free intracellular domain (N-ICD) [11,12]. N-ICD forms a complex with the Suppressor of Hairless (Su(H)) protein, enters the nucleus, and acts a transcriptional cofactor to regulate Notch target gene expression.

In addition to modulating Hh signaling as initially detected by an increase in distance between wing veins L3 and L4 (Fig. 1 A-D), NS1 also had a prominent effect of generating notches along the wing margin (Fig. 1 C,D) indicative of an effect on N signaling. Since N plays a central role in establishing the D/V border of the wing, we next examined whether NS1 had an effect on the expression of various downstream N target genes. We found that, in the presence of NS1(Vn), expression of one such target, Cut, was greatly reduced (Fig. S4 A,B), while another, a *Gbe-lacZ* reporter, was increased (Fig. S4 D,E) analogous to the differing effects on the various Hh target genes. Also, similar to the effect on Hh targets, NS1(PR8) induced a stronger and broader range of activity than NS1 (Vn) on N targets (Fig. S4 G,I). Furthermore, ubiquitously expressing the constitutively active N-ICD transcriptional cofactor in the wing primordium leads to a corresponding ectopic pattern of Cut expression (Fig. S4 K) which is ultimately suppressed by NS1(Vn) (Fig. S4 L). Thus, as in the case of the Hh pathway, NS1(Vn) alters N signaling at the level of its transcriptional effector.

Finally, as with the Hh targets, the A122V mutation reduced the effect of NS1(Vn) (Fig. S4 C,F) and NS1(PR8) (Fig. S4 H,J) on both N targets analyzed suggesting that the same NS1 interaction surface engages both Hh and N transcriptional effectors.

**NS1 activity is independent of Fused kinase, Knot, and Master of Thickveins**

Fused kinase (Fu) is required for converting Ci-155 into an active yet labile transcription factor, which may be the form that interacts with NS1(Vn) [13,14]. We tested whether Fused is required for NS1 activity by expressing NS1(Vn) in *fused* mutant wing discs and analyzed *dpp-lacZ* expression (Fig. S5 C,D). NS1 still augmented *dpp-lacZ* expression in these *fused* mutant discs indicating that NS1 interacts with a form of Ci that is activated independently of *fused*.

There is evidence that Hh, acting via Ci-activation of Knot/Col, induces expression of *master of thickveins* (*mtv*), a gene that can modulate the Dpp response at the A/P border [15,16]. *mtv* encodes a transcription factor that represses expression of the Dpp receptor, Tkv, reducing further the normal low level of Dpp signaling at the A/P border. Since, it has been demonstrated that elimination of Dpp signaling can increase *dpp* expression [17] and because expression of *mtv-lacZ,* like *dpp-lacZ*, is increased by NS1(Vn) (Fig. S5 E,F), we examined whether NS1 activation of *dpp* expression was a consequence of a reduction in Dpp signaling mediated by Knot/Col and/or Mtv. We expressed NS1(Vn) in clones of cells mutant for *mtv* (Fig. S5 G,G`) or in discs mutant for *kn* (Fig. S5 H,I) and found that NS1(Vn) was still able to upregulate the expression of *dpp-lacZ*. We conclude that the ability of NS1(Vn) to augment *dpp* expression is independent of the Knot/Col/Mtv auto-regulatory circuit. The absence of input from the Fused and Knot/Col/Mtv pathways suggest that NS1 acts by regulating the activity of Ci^act^ itself, perhaps bychanging its specific activity.

**The NS1 A122V mutation does not alter temporal regulation of influenza genes**

We considered the possibility that PR8-A122V accelerates activation of the viral gene expression program since A122 lies in a region of NS1 that has been implicated in temporal regulation of viral gene expression [6]. We found no differences, however, in the levels of the influenza NP or M proteins produced in A549 human lung epithelial cells infected with the PR8-WT versus PR8-A122V viruses 4 or 8 hours post infection (Fig. S7A). We conclude that temporal regulation of viral genes is unaltered by A122V, and thus is not the cause for the hastened lethality in PR8-A122V infected mice.

**Materials and Methods:**

**Cloning and Site-directed mutagenesis**

cDNAs for *Drosophila* germline transformation and tissue-culture cell transfection were inserted into Gateway Technology vectors (Invitrogen). Point mutations were made with the QuikChange Site-Directed Mutagenesis Kit (Stratagene), and products were sequenced in their entirety before recombination into destination vectors.

*Viral NS1 constructs:* The cDNAs of NS1 from A/Vietnam/1203/04 (Vn; provided by Robert Webster), A/Udorn/72 (Ud), A/California/09 (Sw), and A/Puerto Rico/8/1934 (PR8) were inserted into pENTR-SD/TOPO (Invitrogen) via “topo cloning”. The destination vector used for germline transformation into *w^118^* flies is pTFHWM, which contains pUASt regulatory elements and encodes both a composite N-terminal Flag/HA epitope tag and a C-terminal Myc epitope tag (modified from plasmids available at the DGRC). At least 3 individual *Drosophila* lines were analyzed for each transformed construct.

*CPSF30 protein expression construct:* An N-terminal fragment of CPSF30 encompassing four zinc finger domains (1-F4) was inserted in pENTR-SD/TOPO by “topo” cloning then recombined into destination vector, pcDNA-DEST40 (Invitrogen) which has a C-terminal V5 epitope tag and promoter elements required for expression in HEK293T cells.

*NS1 construct for bacterial expression:* pGEX3X-NS1/Ud 1-215 was made as described in [18] and purified using glutathione-sepharose beads.

*NS1 construct for NL20 cell expression:* cDNA of PR8 NS1-WT was inserted into pENTR-SD/TOPO by “topo” cloning then recombined into destination vector, pcDNA-DEST40 (Invitrogen) which has promoter elements required for mammalian cell expression. The mutant construct was generated with Stratagene Site-Directed Mutagenesis Kit.

**Western blotting**

*In vitro GST pull-down assay*

200 ng of purified GST or GST-NS1(Ud) was bound to glutathione Sepharose beads in TBS+0.5% NP40 for 1 hr at 4°C in a volume of 100 μl. Beads were washed twice and then incubated with 400 ul of a HEK293T cell lysate containing a V5 tagged N-terminal fragment of CPSF over night at 4°C. Beads were then washed three times and eluted with 2X SDS-PAGE loading buffer and boiled for 5 min prior to loading. Proteins were visualized by Western blotting with mouse anti-V5 (Invitrogen) and rabbit anti-GST primary antibodies. Alexaflour-488 and -647 were used as secondary antibodies and visualized with a Typhoon 9400 scanner.

*Detection of NS1 from fly extracts*

Flies carrying UAS-NS1 constructs were crossed to flies carrying a heat shock-GAL4 driver. 10 progeny flies were then heat shocked at 37°C for 2 hours and allowed to recover for 1 hour. Flies were then homogenized in PBST, centrifuged, and supernatants recovered. SDS-PAGE loading buffer and reducing agent were then added to the supernatant and boiled for 5 minutes prior to loading on an SDS-PAGE gel. Following Western blotting, the NS1 protein was visualized with mouse anti-Myc (Sigma, 1:2000) and/or rabbi anti-HA (AbCam, 1:1000) antibodies. Alexaflour-488 and -647 were used as secondary antibodies and visualized with a Typhoon 9400 scanner.

*Detection of viral proteins from infected A549 cells*

A549 cells were grown in DMEM supplemented with 10% heat inactivated fetal bovine serum (FBS). Cells were infected with 5 plaque forming units (pfu/cell) of the indicated virus. After 1 hour of adsorption at 37°C, cells were washed once with PBS and replenished with DMEM containing 2% FBS and incubated at 37°C for the indicated times. Cells collected at the indicated times after infection were lysed in RIPA buffer (50mM Tris-HCl pH 7.5, 150mM NaCl, 1% NP-40, 0.5% sodium deoxycholate, 0.1% SDS) supplemented with Complete® protease inhibitor (Roche). Immunoblots were probed using the following primary antibodies: rabbit antibody against the major structural proteins of the Ud virus, which detects the nucleocapsid (NP) and matrix (M1) proteins provided by Robert A. Lamb [19] and rabbit anti β-tubulin antibody (Cell Signaling).

**Antibodies used for immunohistochemistry of Drosophila wing discs, mouse lungs, and NL20 cells**

Primary antibodies used for staining *Drosophila* wing discs are mouse anti-beta-galactosidase 1:20 (DSHB), Rabbit anti phospho SMAD1 1:1000 (P.Ten Dijke), rabbit anti-myc 1:100 (Sigma), rabbit anti-Spalt 1:500 (Jose De Celis), mouse anti-patched 1:200 (DSHB), mouse anti-collier 1:100 (Crozatier), rat anti-Ci 2A1 1:2 (Holmgren), mouse anti en 1:5 (DSHB), and mouse anti cut 1:100 (DSHB). Alexaflour secondary antibodies (Invitrogen) were used at 1:1000. Antibodies used for mouse lung immunohistochemistry were rabbit anti-NS1 1:500 (ThermoScientific, PA5-32243), goat anti-Ptc1 1:50 (C-20, Santa Cruz Biotechnology sc-6147), and goat anti BMP-2 1:50 (A-20, Santa Cruz Biotechnology sc-6267). Secondaries used were Alexa Fluor 555 donkey anti-rabbit IgG 1:500 (Life technologies A31572) and Alexa Fluor 488 chicken anti-goat IgG 1:500 (Life technologies A21467). The NS1 and BMP2 antibodies were also used to stain NL20 cells.

**Quantification of viral titers in mouse lungs**

Lungs were homogenized in MEM + 0.1% bovine serum albumin (BSA) by bead beating. Homogenate was 10-fold serially diluted and 500ul was added to MDCK cell monolayers in 6-well plates. Following incubation for 1 hour at 37^0^C, virus was removed and cells were overlaid with 3 ml of a 1:1 MEM:agarose solution containing 1ug/mL TPCK-treated trypsin. After 48 hours, cells were fixed with 4% PFA and stained with a 0.1% solution of crystal violet and plaques were counted.

**Confocal imaging and quantification**

Confocal images of wing imaginal discs were acquired on a Leica SP2 using a 40X/1.3NA oil objective. Confocal images of NL20 cells and mouse lung tissue were obtained on a Leica SP8 equipped with HyD detectors using a 40X/1.3NA oil objective. All intensity and colocalization quantification was done using Imaris software (Bitplane).

**FRET-FLIM imaging**

Using a Leica SP5 with a 40X/1.3NA objective, fluorescence resonance energy transfer by fluorescence lifetime imaging (FRET-FLIM) was carried out on wing imaginal discs stained with Ci-155 and NS1 primaries and Alexa-488 and Alexa-555 secondaries, respectively. Analysis of fluorescence decays were resolved by time-correlated single-photon counting (TCSPC) using an SPC830 acquisition board (Becker & Hickl, Berlin, Gremany). Two-photon excitation of Alexa 488 fluorophore was performed at 800 nm by a femtosecond mode-locked (80-MHz repetition rate) Mai-Tai HP pulsed multiphoton laser (Spectra Physics). Images were acquired in 256- by 256-pixel format, collecting in excess of 1,000 photons per pixel in 2 to 5 min, and the fluorescence transients were acquired by using SPCIMAGE software (Becker & Hickl, Berlin, Germany). The results were exported and analyzed with an in-house-developed image analysis protocol using Image J NIH imaging software as previously described [20]. FRET efficiency was calculated using the formula:

F_eff_=1-(Tau_DA_/Tau_D_), where Tau_DA_ is the fluorescence lifetime of the donor in the presence of the acceptor, and Tau_D_ is the lifetime of the donor alone.

**Statistics**

P-values of signal intensities from confocal images, distance measurements in adult wings, and cytokine levels were calculated in Excel using a two-tailed, homoscedastic Student’s T-test. The p-value of mouse lethality was calculated using the log rank Mantel-cox test. P-values less than 0.05 were considered significant.

**Drosophila genetics and screens**

*Fly crosses*

*dpp*-*lacZ*, *mtv-lacZ, or Gbe-lacZ* (which contains several Su(H) promoter binding sites) expression in discs expressing NS1, was analyzed by crossing females with the genotype *w; dpp-lacZ; 71B* or *w; dpp-lacZ; C765*, or *dpp-lacZ^EP^;* 71B; or *ptc-GAL4; dpp-lacZ*, or *w; mtv-lacZ; 71B,* or *dpp-lacZ; 71B CiRNAi,* or, *w* *Gbe-lacZ; C765*  to w; *UAS-NS1* males or mutant variants. *dpp-lacZ* encodes the dpp disk enhancer described in [21]. *dpp-lacZ^EP^* is an enhancer trap reporter of *dpp* expression (provided by Daniel Kalderon). *dpp* expression was assayed in: **1)** discs expressing components of the Hh, Dpp signaling pathways with or without NS1 by crossing females with the genotype *UAS-Tkv* or *UAS-Tkv^DN^* or *UAS-Sog^CR1^* or *UAS-PKA^act^* *or UAS-PKA^R^* or *UAS-smoD1-3* or *UAS-Ptc* or *UAS-Ci^S849A^* to males with the genotype *w; dpp-lacZ; 71B* or *w; UAS-NS1 dpp-lacZ/CyO; 71B/TM6*; **2)** discs expressing Gli1 with or without NS1, by crossing females with the genotype *w; dpp-lacZ; 71B* or *w; dpp-lacZ^EP^/CyO; 71B/TM6B* to males of the genotype *w; UAS-Gli1/TM6B* or *w; UAS-NS1/CyO; UAS-Gli1/TM6B;* **3)** in *fu* mutant discs was analyzed by crossing females with the genotype *ywhsflp fu^mH63^; FRTDy+175[fu]/CyO; 71B/TM6B* to males with the genotype *yw; UAS-NS1; dpp-lacZ* and *y-* males were selected; **4)** in *mtv* mutant clones expressing NS1 was examined by crossing females of the genotype *ywhsflp; FRT40Aubi-GFP; 71B* to males of the genotype *w; FRT40Amtv^6^/CyO; UAS-NS1 dpp-lacZ*; and **5)** in *kn* mutant discs expressing NS1 by crossing females of the genotype *w; kn^1^; 71B* to males with the genotype *w; kn^1^; UAS-NS1* *dpp-lacZ*. Finally, we analyzed *dpp* expression dependent upon NS1 with or without co-expression of Ci^S849A^ in *cos-2* mutant clones by crossing females of the genotype *ywhsflp; 42Bubi-GFP; 71B dpp-lacZ* to males of the genotype *w; 42Bcos-2^2^/CyO; UAS-NS1* or *w; 42Bcos-2^2^/CyO; UAS-NS1 UAS-Ci^S849A^*. Cut expression was assayed in discs from a cross with females with the genotype UAS-NICD to males with the genotype C765 or UAS-NS1/CyO; C765/TM6B.

*EMS mutagenesis of Drosophila*

Male flies with the genotype *w; UAS-NS1/CyO* were starved for approximately 8 hours and then placed in a vial with a Kim-wipe adsorbed with 1.2mL of a 0.26% EMS (Sigma) solution in 5% sucrose. The flies were allowed to feed on the EMS overnight and then crossed to females with the genotype *w MS1096-GAL4*. Transgenes from progeny displaying a revertant phenotype were sequenced and then verified by cloning the identified mutations into fresh UAS expression vectors and transforming them back into flies, thereby ruling out possible second site mutations.

**References**

1. Nellen D, Burke R, Struhl G, Basler K (1996) Direct and long-range action of a DPP morphogen gradient. Cell 85: 357-368.

2. Yu K, Srinivasan S, Shimmi O, Biehs B, Rashka KE, et al. (2000) Processing of the Drosophila Sog protein creates a novel BMP inhibitory activity. Development 127: 2143-2154.

3. Hale BG, Randall RE, Ortin J, Jackson D (2008) The multifunctional NS1 protein of influenza A viruses. J Gen Virol 89: 2359-2376.

4. Donelan NR, Basler CF, Garcia-Sastre A (2003) A recombinant influenza A virus expressing an RNA-binding-defective NS1 protein induces high levels of beta interferon and is attenuated in mice. J Virol 77: 13257-13266.

5. Nemeroff ME, Barabino SM, Li Y, Keller W, Krug RM (1998) Influenza virus NS1 protein interacts with the cellular 30 kDa subunit of CPSF and inhibits 3'end formation of cellular pre-mRNAs. Mol Cell 1: 991-1000.

6. Min JY, Li S, Sen GC, Krug RM (2007) A site on the influenza A virus NS1 protein mediates both inhibition of PKR activation and temporal regulation of viral RNA synthesis. Virology 363: 236-243.

7. Shin YK, Li Y, Liu Q, Anderson DH, Babiuk LA, et al. (2007) SH3 binding motif 1 in influenza A virus NS1 protein is essential for PI3K/Akt signaling pathway activation. J Virol 81: 12730-12739.

8. Heikkinen LS, Kazlauskas A, Melen K, Wagner R, Ziegler T, et al. (2008) Avian and 1918 Spanish influenza a virus NS1 proteins bind to Crk/CrkL Src homology 3 domains to activate host cell signaling. J Biol Chem 283: 5719-5727.

9. Irvine KD, Vogt TF (1997) Dorsal-ventral signaling in limb development. Curr Opin Cell Biol 9: 867-876.

10. Ito T, Connett JM, Kunkel SL, Matsukawa A (2012) Notch system in the linkage of innate and adaptive immunity. J Leukoc Biol 92: 59-65.

11. Kopan R, Ilagan MX (2009) The canonical Notch signaling pathway: unfolding the activation mechanism. Cell 137: 216-233.

12. Fortini ME (2009) Notch signaling: the core pathway and its posttranslational regulation. Dev Cell 16: 633-647.

13. Ohlmeyer JT, Kalderon D (1998) Hedgehog stimulates maturation of Cubitus interruptus into a labile transcriptional activator. Nature 396: 749-753.

14. Zhou Q, Kalderon D (2011) Hedgehog activates fused through phosphorylation to elicit a full spectrum of pathway responses. Dev Cell 20: 802-814.

15. Crozatier M, Glise B, Vincent A (2002) Connecting Hh, Dpp and EGF signalling in patterning of the Drosophila wing; the pivotal role of collier/knot in the AP organiser. Development 129: 4261-4269.

16. Funakoshi Y, Minami M, Tabata T (2001) mtv shapes the activity gradient of the Dpp morphogen through regulation of thickveins. Development 128: 67-74.

17. Haerry TE, Khalsa O, O'Connor MB, Wharton KA (1998) Synergistic signaling by two BMP ligands through the SAX and TKV receptors controls wing growth and patterning in Drosophila. Development 125: 3977-3987.

18. Melen K, Kinnunen L, Fagerlund R, Ikonen N, Twu KY, et al. (2007) Nuclear and nucleolar targeting of influenza A virus NS1 protein: striking differences between different virus subtypes. J Virol 81: 5995-6006.

19. Chen BJ, Leser GP, Morita E, Lamb RA (2007) Influenza virus hemagglutinin and neuraminidase, but not the matrix protein, are required for assembly and budding of plasmid-derived virus-like particles. J Virol 81: 7111-7123.

20. Ganesan S, Rohde G, Eckermann K, Sroka K, Schaefer MK, et al. (2008) Mutant SOD1 detoxification mechanisms in intact single cells. Cell Death Differ 15: 312-321.

21. St Johnston RD, Hoffmann FM, Blackman RK, Segal D, Grimaila R, et al. (1990) Molecular organization of the decapentaplegic gene in Drosophila melanogaster. Genes Dev 4: 1114-1127.
