# Supplementary figures and images for: Influenza NS1 directly modulates Hedgehog signaling during infection
Source: PLoS Pathog. 2017 Aug 24;13(8):e1006588. doi: 10.1371/journal.ppat.1006588 (PMC5587344; doi:10.1371/journal.ppat.1006588)

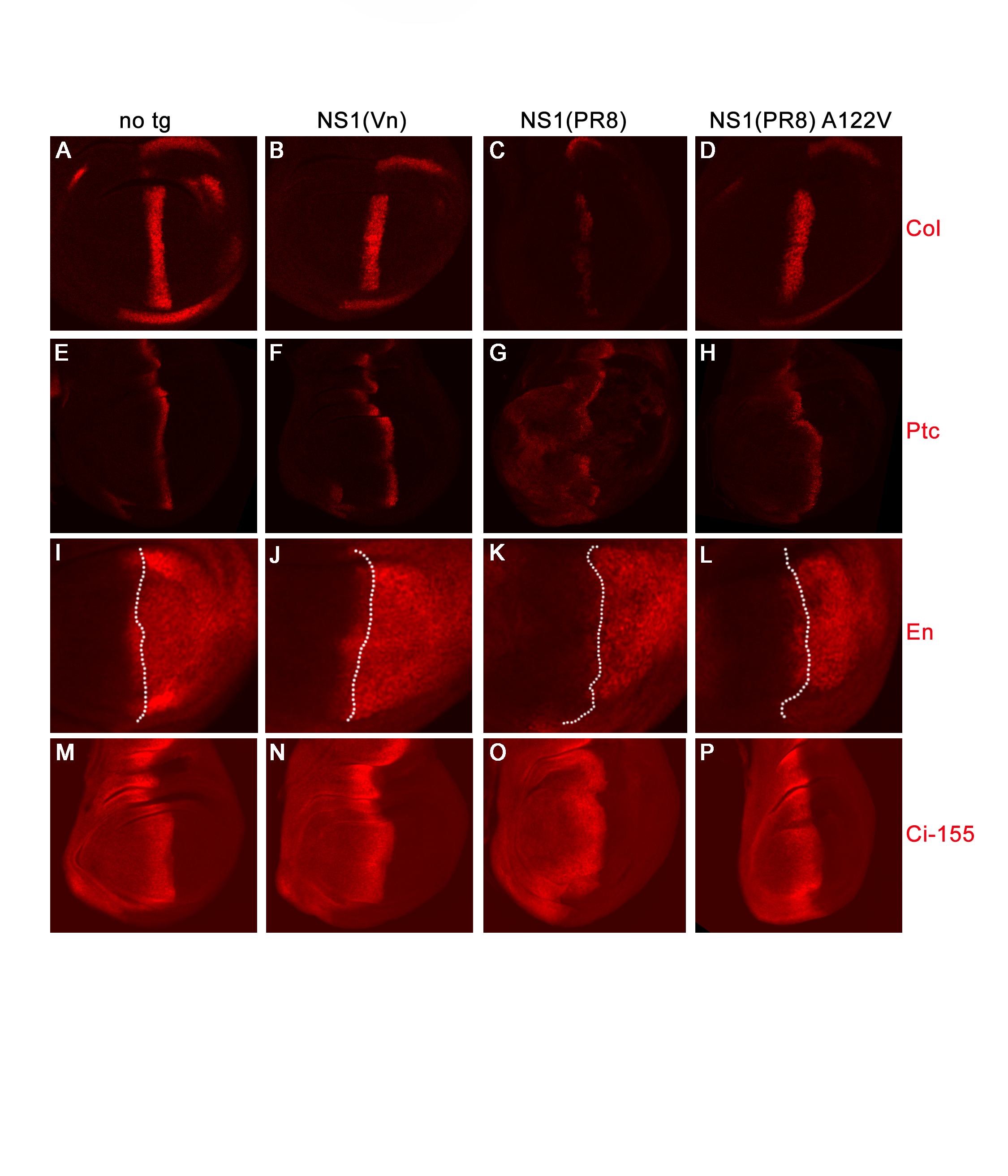

Supplement: S1 Fig — Wing imaginal discs with no transgene (no tg) or ubiquitously expressing the indicated transgenes were stained with antibodies recognizing Collier (Col) (A-D), Ptc (E-H), Engrailed (En) (I-L), and Ci-155 (M-P). The moderately active NS1(Vn) has little, if any, effect on expression Col (B vs. A), Ptc (F vs. E), or Hh-dependent anterior En expression which is present in late third instar wing discs (J vs. I), or full length Ci (N vs. M). The more strongly active NS1(PR8), however, results in reduced expression of Col (C vs. A), ectopic expression of Ptc (G vs. E), and reduced anterior expression of En (K vs. I). Each of these NS1(PR8) activities is greatly attenuated by the A122V mutation (D, H, L). Similar to NS1(Vn), NS1(PR8) does not appreciably alter Ci-155 levels (O,P). White lines in I-L denote the A/P border. (TIF) [file ppat.1006588.s002.tif]

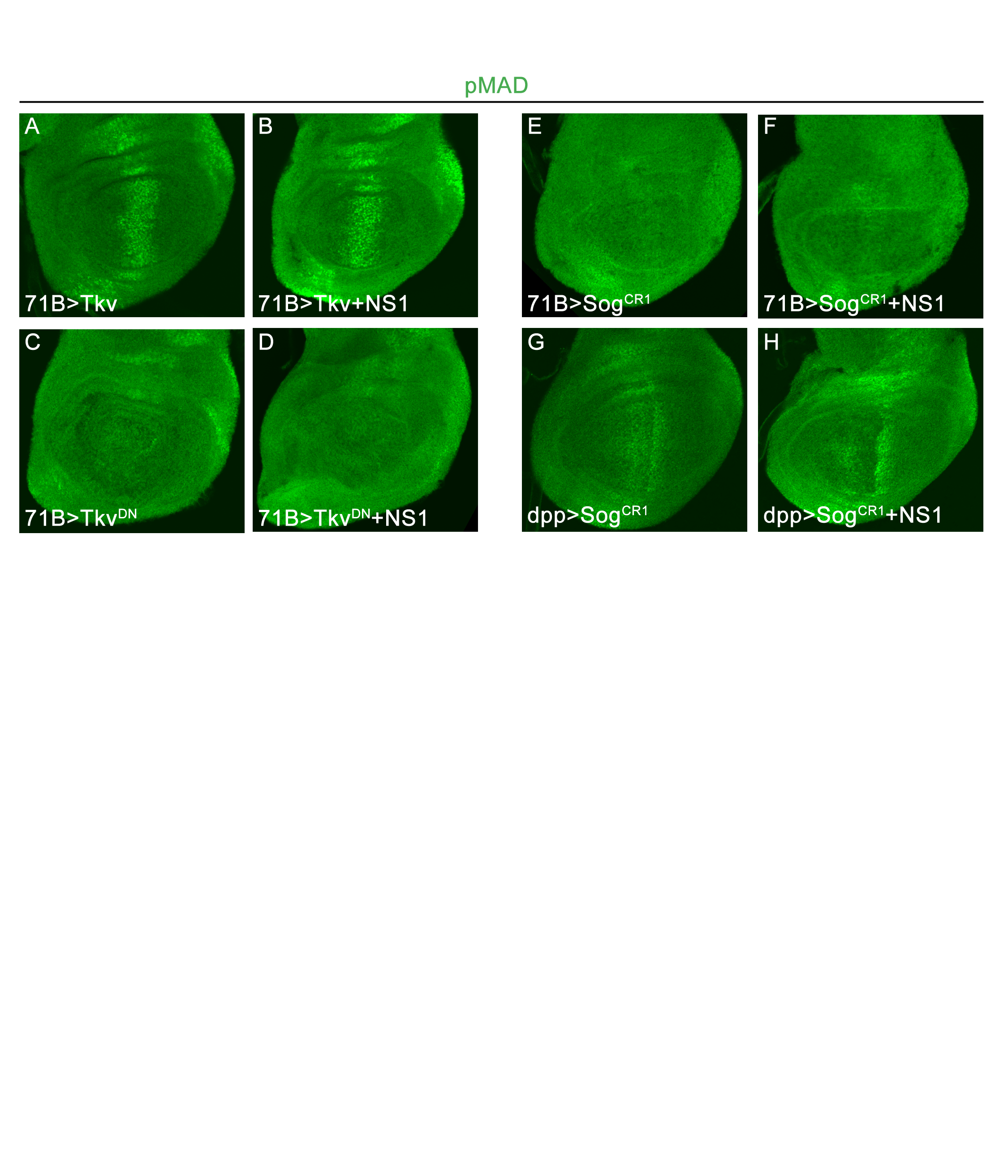

Supplement: S2 Fig — Wing discs expressing the indicated transgenes with the ubiquitous 71B-GAL4 driver (A-F), or along the A/P border with the dpp-GAL4 driver (G, H), were assayed for the pattern of Dpp response by staining with a phospho-Smad1 antibody (pMad). Ubiquitous expression of a wild-type form of the Dpp type-II receptor subunit Thick veins (Tkv) restricts Dpp signaling to the A/P border (A). While pMAD staining is restricted to the A/P border, NS1 activity is not compromised (B). Expression of a dominant negative form of the Tkv receptor subunit eliminates both endogenous and NS1-activated pMAD staining (C, D). Ubiquitous expression of the potent secreted SogCR1 Dpp antagonist (Yu et al., 2000) also results in strong reduction of the NS1 response (E, F), while A/P border expression of SogCR down-regulates the response strongly in A/P border cells but seems to allow some signal to escape into adjacent regions which can be enhanced by NS1 (G, H). (TIF) [file ppat.1006588.s003.tif]

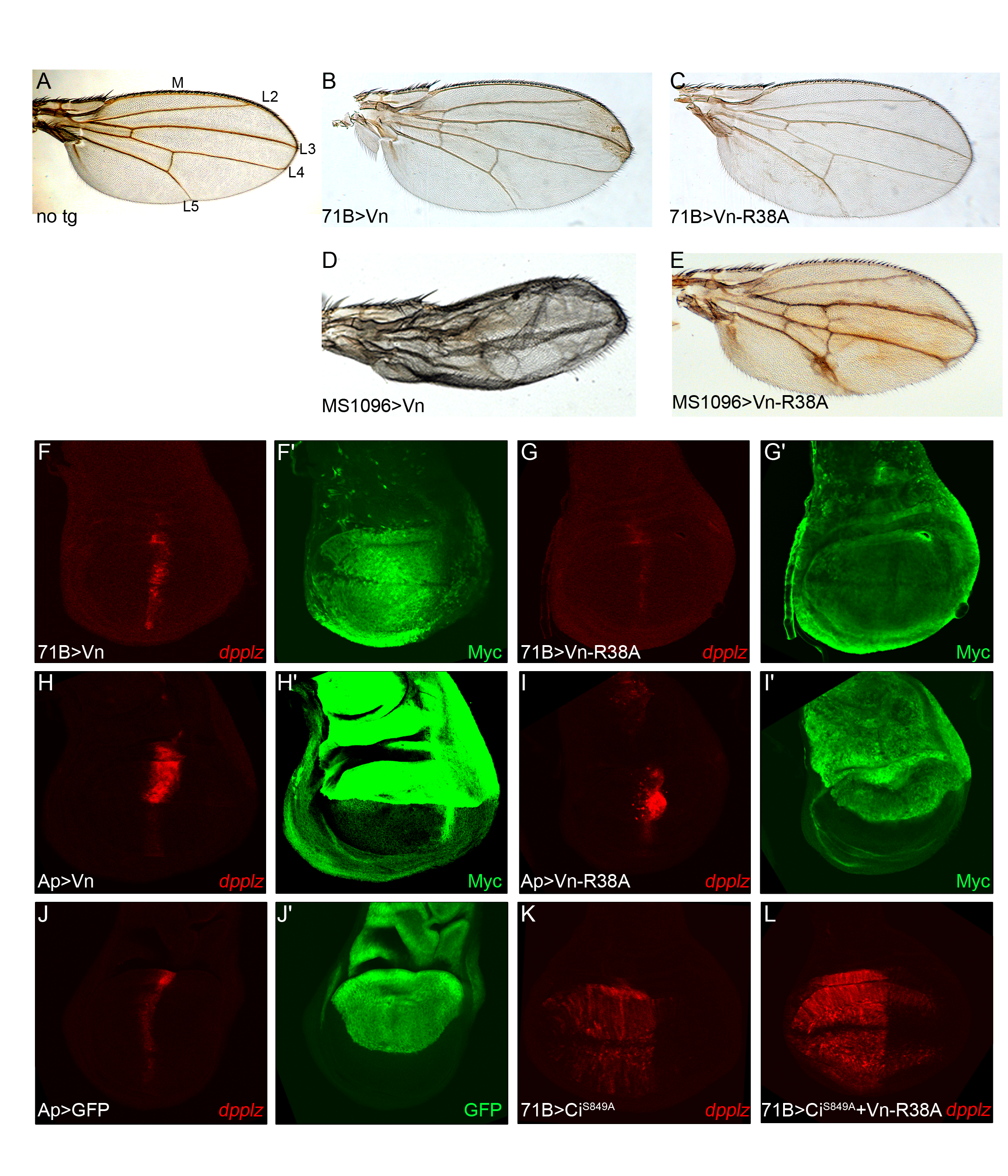

Supplement: S3 Fig — (A) A wing with no transgene (no tg) with demarcated longitudinal veins L2-L5, M = margin. Wings expressing NS1(Vn)-R38A (C,E) have a reduced phenotype compared to wings expressing wild type NS1(Vn) (B,D) with either the moderate wing GAL4 driver, 71B (B,C) or the stronger driver, MS1096 (D,E). (F-L) Wing discs expressing the indicated transgene were assessed for NS1 activity by measuring dpp-lacZ expression (F,G,H,I,J,K,L). NS1 protein levels were also analyzed by staining discs with a Myc antibody directed towards the C-terminal tag of the transgene (F'.G',H',I'). NS1(Vn)-R38A appears to have lower protein levels (G' vs. F' and I' vs H') and less ability to activate dpp-lacZ expression when expressed with 71B-GAL4 (G vs. F). However, when expressed with the stronger driver, Apterous-GAL4 (Ap) which is expressed only in the dorsal compartment of the wing disc (green area in J'), NS1(Vn)-R38A is able to enhance dpp-lacZ expression in this domain (I) compared to discs expressing a GFP construct (J) and to similar levels as NS1(Vn) (H). NS1(Vn)-R38A is also able to enhance CiS849A-mediated ectopic expression of dpp-lacZ (L vs. K). (TIF) [file ppat.1006588.s004.tif]

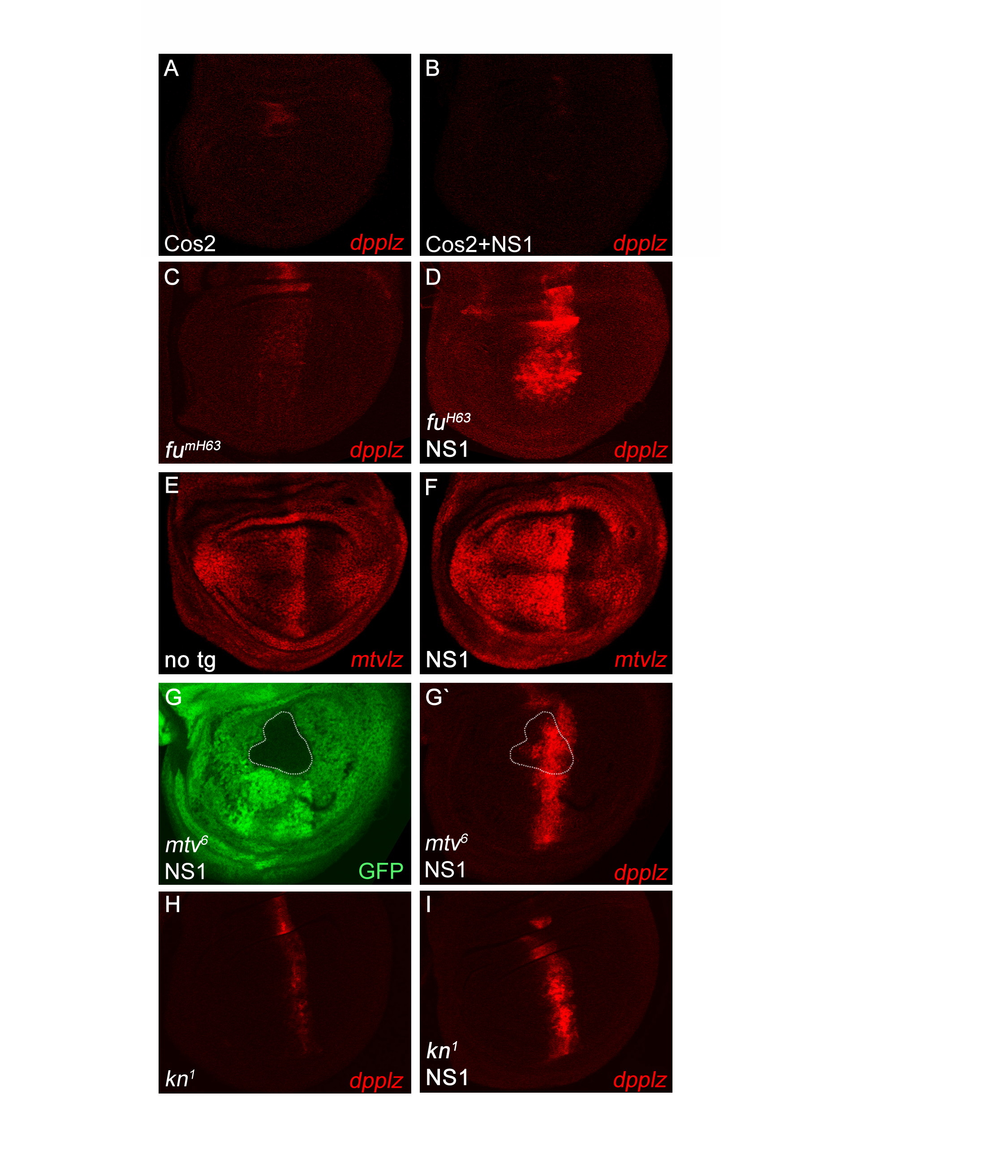

Supplement: S4 Fig — Reducing the levels of Ci-155 by ubiquitously expressing Cos-2 blocks endogenous dpp-lacZ expression (A), and also blocks the ability of NS1 to enhance dpp-lacZ expression (B). In fused (fumH63) mutant discs, dpp-lacZ expression is broadened but not elevated (C). Expression of NS1 in a fu mutant background can augment dpp-lacZ expression throughout the expanded response zone (D), indicating that fu is not required for the dpp-activating function of NS1, although Fused is required for full activation of Ci-155. NS1(Vn) can enhance mtv-lacZ expression at the A/P border and activate expression throughout a broad domain in the anterior compartment (F vs. E). In clones of cells expressing a strong hypomorphic allele of mtv (mtv6, marked by the absence of GFP in G and outlined in G and G`), high NS1-dependent expression of dpp-lacZ is unaffected (G`). In discs expressing a hypomorphic allele of kn (kn1), expression of the dpp-lacZ reporter construct is not significantly altered (H), nor is the ability of NS1(Vn) to elevate expression of this reporter (I). Where present, transgenes were expressed with the ubiquitous 71B-GAL4 driver. (TIF) [file ppat.1006588.s005.tif]

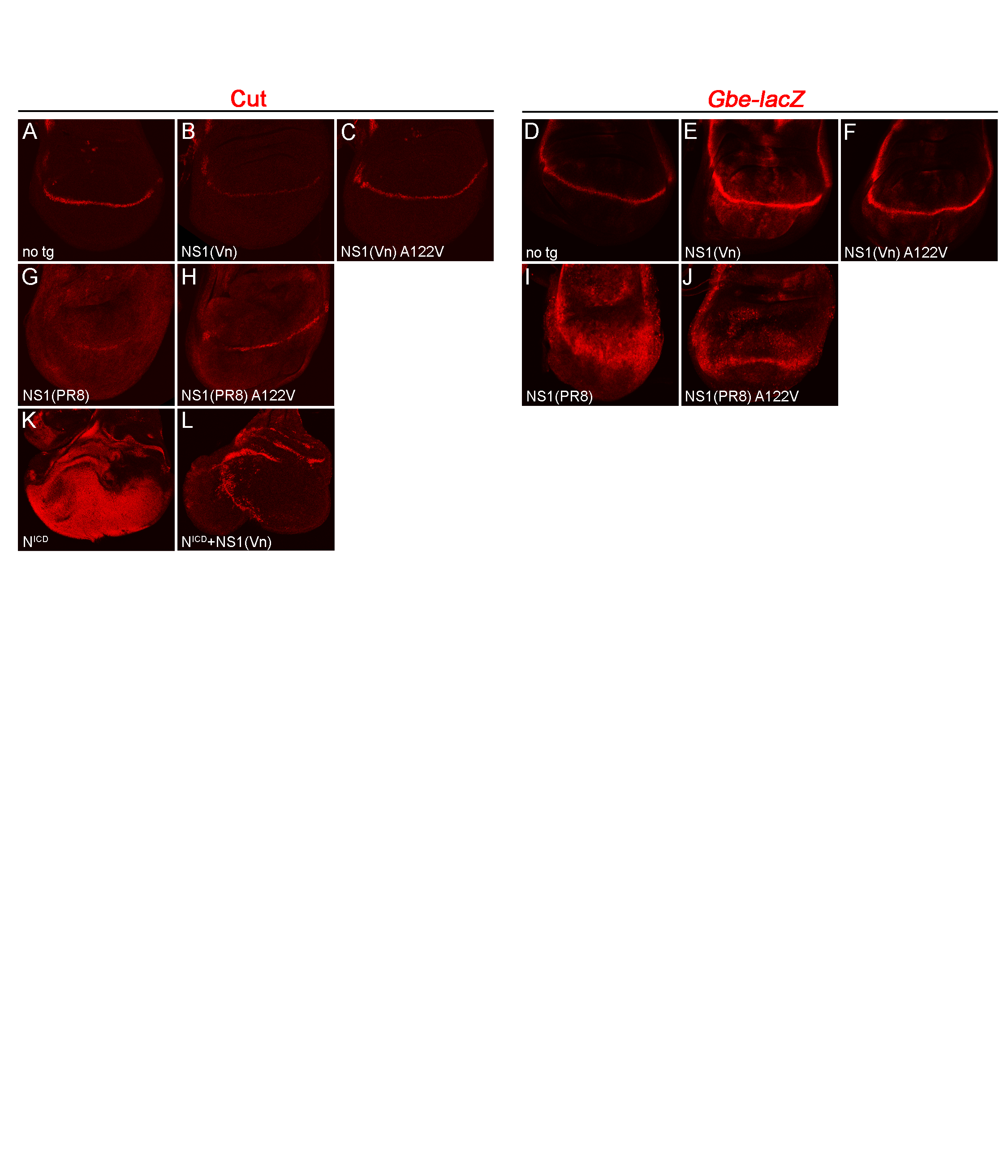

Supplement: S5 Fig — NS1 (Vn) reduces expression of Cut along the presumptive wing margin (B vs. A) and increases expression of Gbe-lacZ, a synthetic Notch reporter gene construct (E vs. D). Both of these effects of NS1 (Vn) are greatly reduced by the A122V mutation (C vs. B and F vs. E). The effect of NS1 (PR8) is stronger than NS1 (Vn) with regard to Cut repression (G vs. B) and Gbe-lacZ activation (I vs. E), and both of these effects are reduced by the A122V mutation (H vs. G and J vs. I, respectively). NS1(Vn) also blocked ectopic expression of Cut induced by ubiquitous expression of the activated N-ICD transcriptional effector (L vs. K). (TIF) [file ppat.1006588.s006.tif]

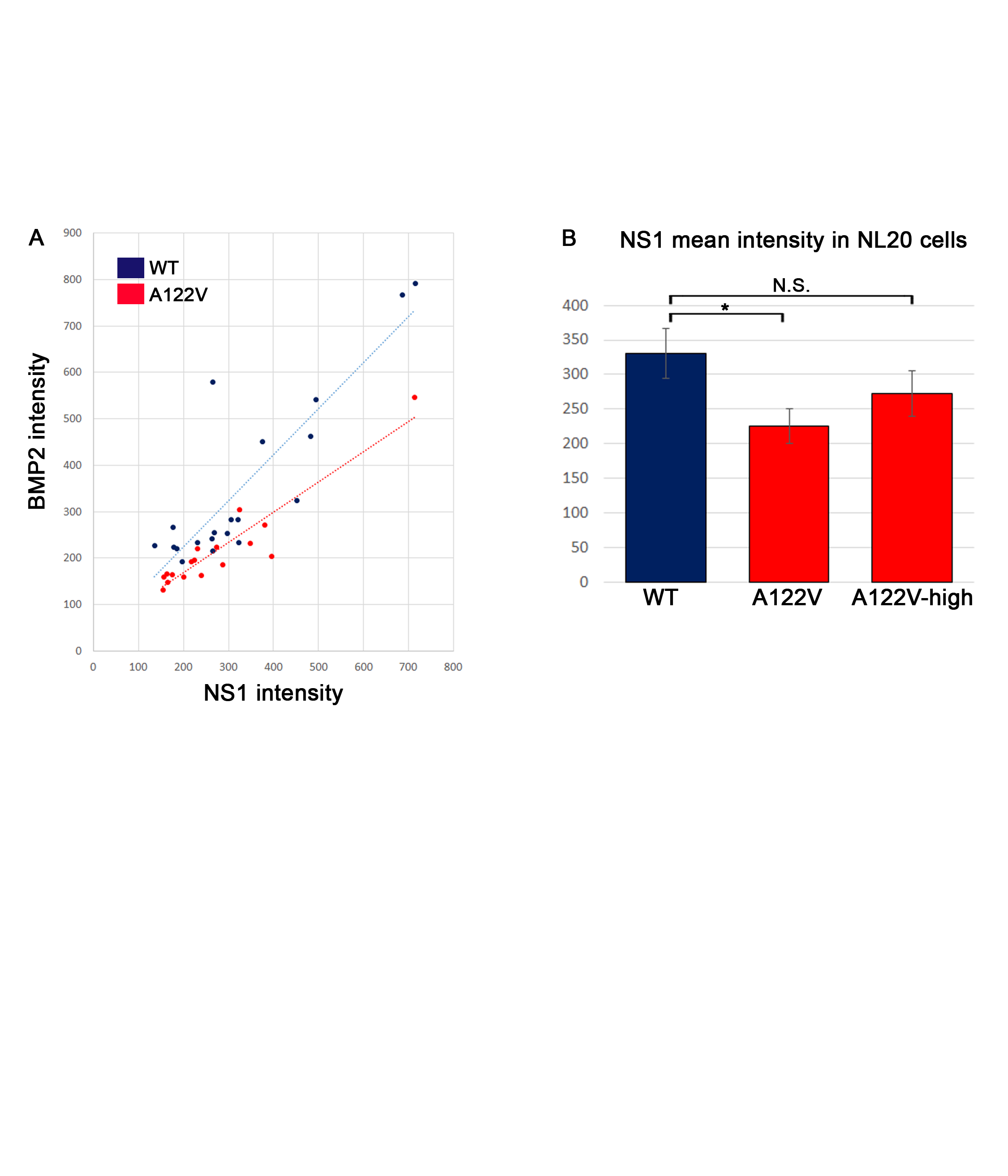

Supplement: S6 Fig — (A) NL20 cells transfected with the indicated NS1-expressing plasmids show an induction of BMP2 expression proportional to NS1 expression. (B) The mean intensity of NS1 was quantified from 17–20 cells per group (*p = 0.021). (TIF) [file ppat.1006588.s007.tif]

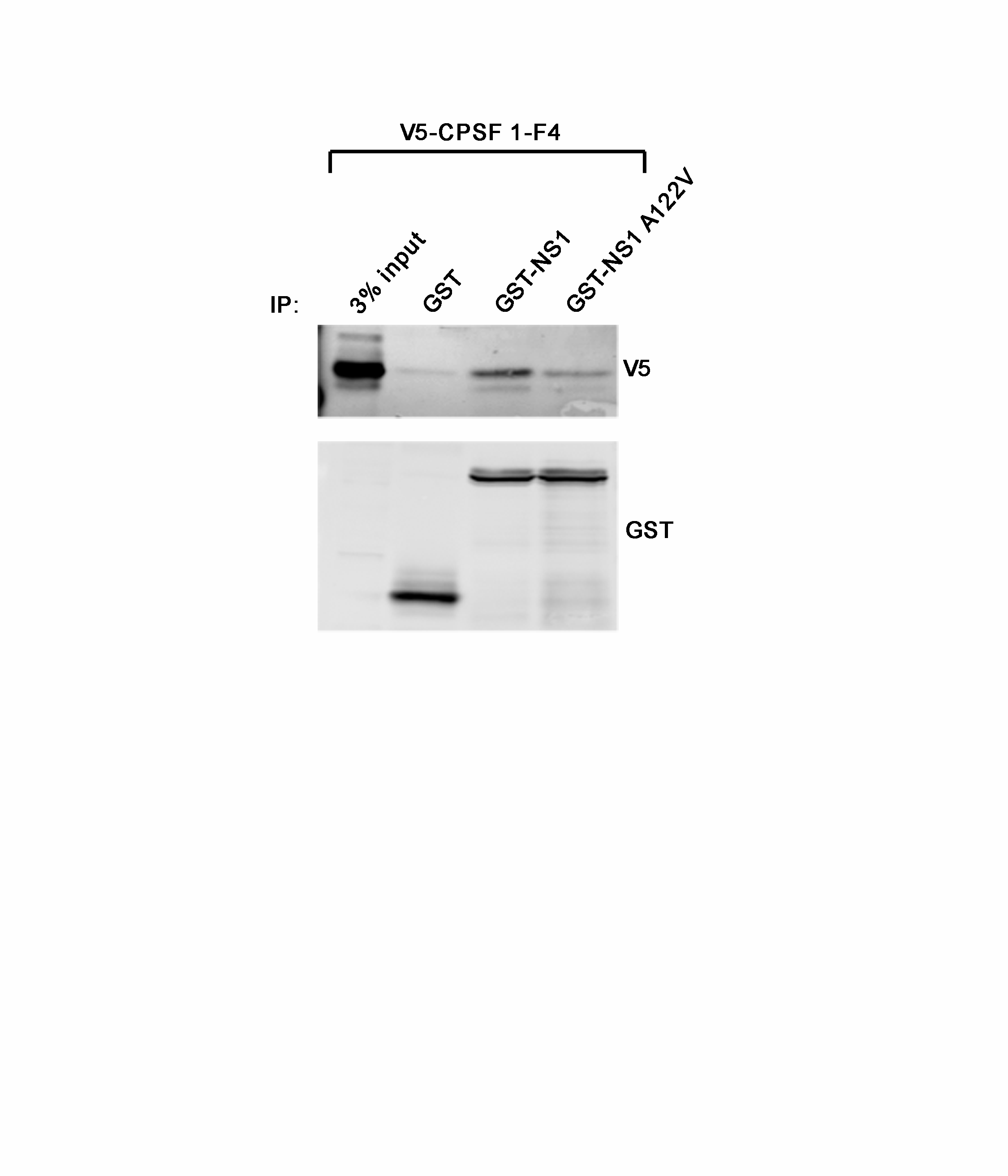

Supplement: S7 Fig — GST or GST-NS1 fusion proteins were incubated with extracts of 293T cells transfected with an N-terminal fragment of CPSF30 containing four of its zinc finger binding domains and tagged C-terminally with a V5 epitope. Proteins immune precipitated with glutathione beads were visualized on Western blots with anti-V5 (top) and anti-GST (bottom) antibodies. The A122V mutation reduces NS1 binding to CPSF30 under these in vitro binding conditions. (TIF) [file ppat.1006588.s008.tif]

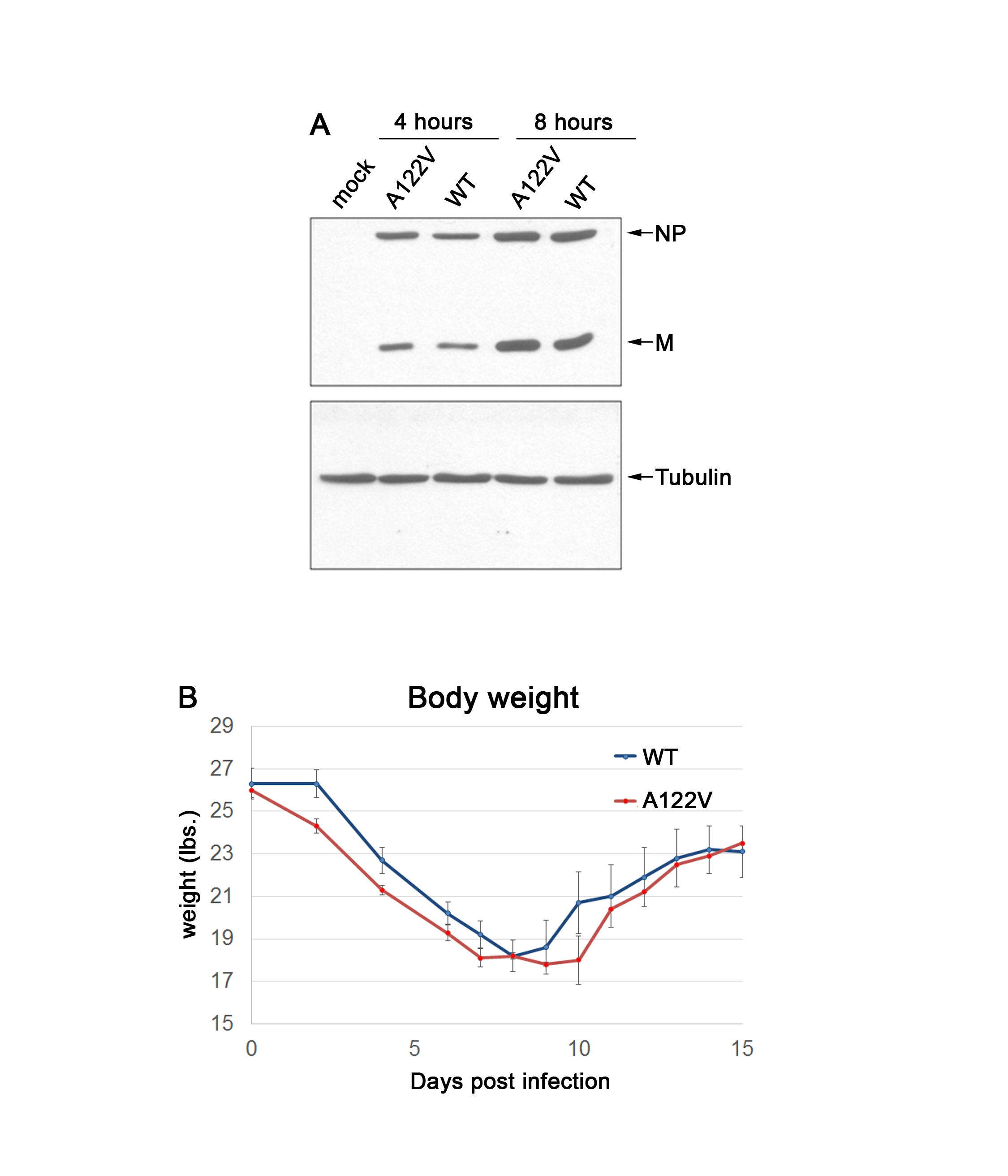

Supplement: S8 Fig — (A) Temporal synthesis of two viral proteins, NP and M, was examined in A549 cells following a high-MOI single cycle infection with the PR8-WT and PR8-A122V viruses. No differences in the levels or kinetics of these markers were observed indicating that the A122V mutation does not alter temporal regulation of viral gene expression. (B) 10 infected mice per group were monitored for weight loss for 15 days. There was no significant difference in weight loss between PR8-WT and PR8-A122V infected animals. (TIFF) [file ppat.1006588.s009.tiff]

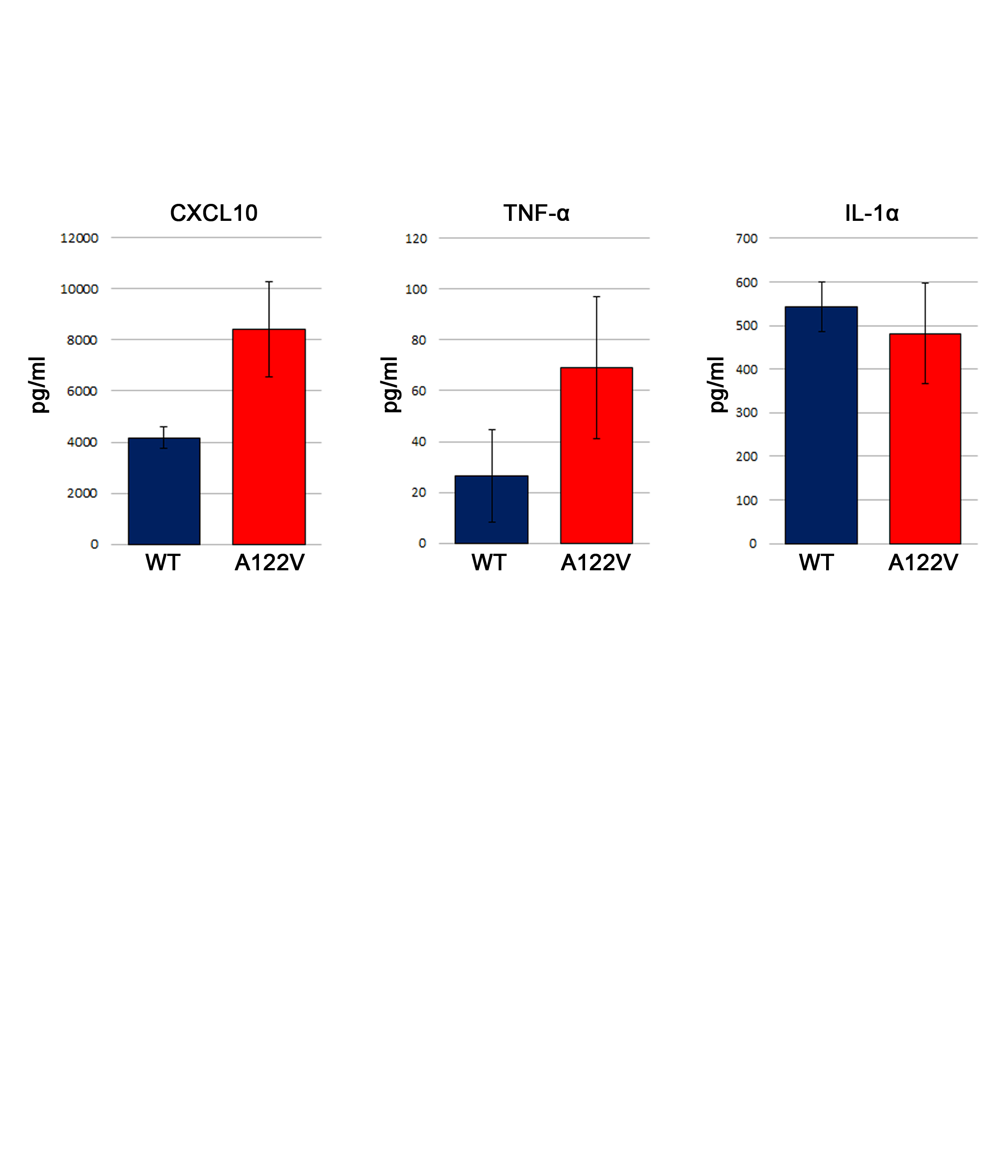

Supplement: S9 Fig — CXCL-10, TNF-α, and IL-1α levels were measured from extracts of mouse lungs infected with PR8-WT and PR8-A122V. CXCL-10 was significantly increased in mutant infected lungs compared to the WT (p = 0.043), whereas TNF-α, and IL-1α were not appreciably changed. 4–5 lungs were analyzed per group. (TIF) [file ppat.1006588.s010.tif]
